# Scaling Up Gas–Liquid Photo-Oxidations in Flow Using Rotor-Stator Spinning Disc Reactors and a High-Intensity Light Source

**DOI:** 10.1021/acs.oprd.4c00458

**Published:** 2025-01-14

**Authors:** Arnab Chaudhuri, Wouter F.C. de Groot, Jasper H.A. Schuurmans, Stefan D.A. Zondag, Alessia Bianchi, Koen P.L. Kuijpers, Rémy Broersma, Amin Delparish, Matthieu Dorbec, John van der Schaaf, Timothy Noël

**Affiliations:** 1 Department of Chemical Engineering and Chemistry, Sustainable Process Engineering, University of Technology (TU/e), Eindhoven 5612 AZ, The Netherlands; 2 Flow Chemistry Group, Van’t Hoff Institute for Molecular Sciences (HIMS), Universiteit van Amsterdam (UvA), Amsterdam 1098 XH, The Netherlands; 3 Technology and Engineering Group, Janssen Research and Development, Turnhoutseweg 30, Beerse 2340, Belgium; 4 Signify Research, Eindhoven 5656 AE, The Netherlands

**Keywords:** scale-up, process intensification, photochemistry, reactor development, photocatalysis

## Abstract

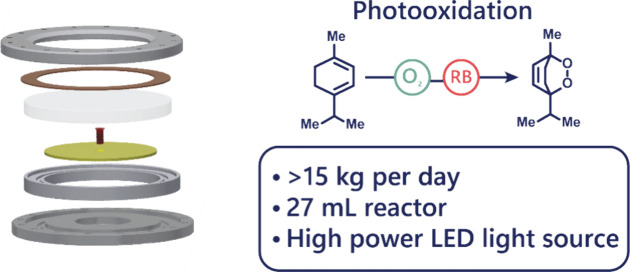

Photochemical transformations have garnered renewed interest over the past decade for their ability to enable unique reactions under mild conditions. However, scaling up such processes, particularly in multiphase systems (e.g., gas–liquid), remains challenging. Previously, we demonstrated the potential of the photochemical rotor-stator spinning disc reactor (pRS-SDR) for scaling the photooxidation of α-terpinene to ascaridole, though the system was limited by the light source, resulting in suboptimal operation in a photon-limited regime. In this work, we unlock the full potential of the pRS-SDR by integrating a high-powered light source (up to 652 W optical output) specifically designed for the reactor. The results show that the high gas–liquid mass transfer rates achievable in the pRS-SDR allow for significant productivity improvements under high irradiance (16.3 kg day^–1^ at 92% α-terpinene conversion and 2.52 W cm^–2^ in a 27 mL irradiated volume), representing an order of magnitude increase compared to our previous study. However, the photooxidation of β-citronellol exhibited notable limitations, highlighting the importance of selecting appropriate model reactions when evaluating intensified photochemical reactors.

## 1 Introduction

Photon activation of molecules presents valuable opportunities for developing new chemical transformations, especially for the pharmaceutical and agrochemical industries.^1,2^ Photochemistry enables reactions to occur under ambient temperatures and pressures, often unlocking pathways that are inaccessible through conventional thermochemical methods.^3−5^ This advantage has led to a resurgence of interest in photochemistry, with recent studies highlighting a wide range of unique chemical transformations.^6−14^ Despite its promise, significant challenges remain in scaling photochemical processes, especially due to the attenuation of light within absorbing media, which can cause uneven photon distribution throughout the reactor.^15^ As a result, conventional large-volume batch reactors are often inefficient for scaling up photochemical reactions due to their poor photon utilization.

In multiphase systems (e.g., gas–liquid or liquid–solid mixtures), mass transport between phases often acts as a rate-limiting step. Multiphase photochemical reactions, such as gas-phase oxidations^12^ or carbonylations,^16^ are important transformations due to their ability to functionalize key compounds with high atom-efficiency.^14^ Scaling up these reactions in conventional batch reactors presents challenges, particularly in mass transport, due to the low energy dissipation rates per volume. This turbulent energy dissipation is a crucial factor influencing mass transfer efficiency.^17^ Therefore, achieving comparable productivity in multiphase photochemical reactions as in traditional processes requires careful design of both the reactor and the light source.

In recent years, various studies have explored technological solutions to the scaling challenges in photochemistry, focusing on continuous-flow reactor implementation and improved light source integration.^18−38^ A key focus has been enhancing mass transfer, achieved through reducing the diffusional path length between phases or intensifying fluid dynamics to increase phase refreshment rates. Microreactor technology, in particular, has shown promising results by further reducing light pathlengths. However, industrial adoption of photochemical processes remains limited, with productivity generally restricted to lab-scale levels.^39,40^ Consequently, recent industry-led studies have shifted their focus toward achieving kilogram- to multikilogram-scale productivity in photochemical reactions.^41−59^

In our previous work, we demonstrated the photochemical rotor-stator spinning disc reactor as a novel technology capable of both intensified mass transfer and high productivity, addressing two key challenges in photochemical reactor design.^60−62^ In this current study, we further develop this technology by integrating a state-of-the-art high-intensity light source, significantly enhancing the reactor’s productivity. We highlight the critical role of gas–liquid mass transfer intensification, particularly under high irradiance conditions, achieving over an order of magnitude improvement in projected productivity within the same compact reactor compared to our earlier results. Notably, for one of our model reactions, we observed the reaction rate to be independent of photon flux density, suggesting inherent limitations within the system and underscoring the importance of selecting appropriate model reactions for such studies.

### 1.1 Photochemical Rotor-Stator Spinning Disc Reactor (pRS-SDR)

**Figure 1:**
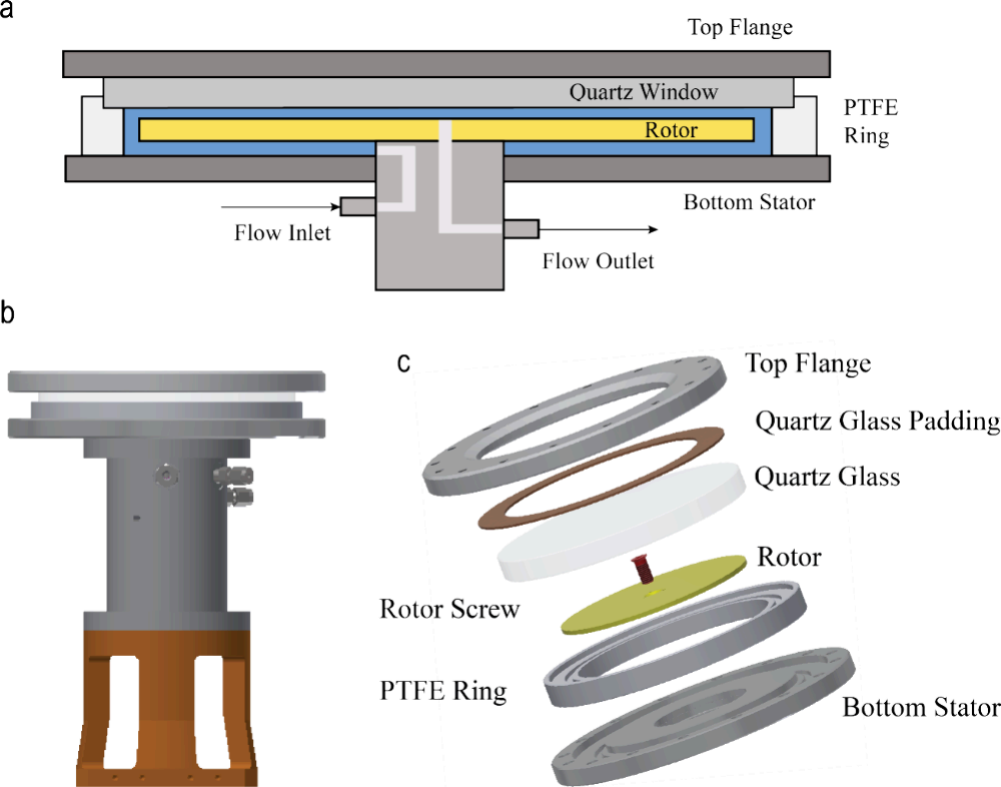
Overview of the photochemical rotor-stator spinning disc reactor (pRS-SDR). (a) Cross-sectional view of the reactor denotating the various components of the system. The section in blue represents the rotor-stator cavity where the reaction medium is located. Only the top section of the reactor is illuminated. (b) 3D model of the reactor including the shaft where the motor is connected to the device. (c) Exploded 3D view of the reactor denoting the various components in the system.

The rotor-stator spinning disc reactor utilizes high energy dissipation rates to enhance mass and heat transfer. The reactor’s design features a high-speed rotating disc within a rotor-stator cavity, with a narrow gap of just a few millimeters (a gap of 2 mm was used in this study), generating significant shear forces.^63^ In gas–liquid flows, these forces create small bubbles and improve dispersion in the liquid phase, leading to high surface-area-to-volume ratios and effective mass transfer between phases.^64^ The high energy dissipation also increases refreshment rates, further enhancing gas–liquid mass transfer. In addition to mass transfer, the reactor’s intensified heat transfer and mixing rates contribute to overall process efficiency.^65−68^ This reactor has previously demonstrated the intensification of multiphase reactions where mass and heat transfer are critical.^69−77^ For the current photochemical application, the stator’s top face has been replaced with a quartz glass plate, allowing light to irradiate the reaction medium, as opposed to the stainless-steel material used in other applications. The reactor has a total volume of 64 mL, with the irradiated portion comprising 27 mL. A schematic of the reactor is shown in Figure 1, and further details are available in the Supporting Information.

In our previous work, we successfully demonstrated the use of the pRS-SDR for scaling gas–liquid^61^ and gas–liquid–solid photochemical reactions.^62^ Although we achieved high productivity in the gas–liquid photochemical oxidation of α-terpinene (1.1 kg day^–1^ of ascaridole), the light source proved to be a key limiting factor.^61^ To address this, we collaborated with researchers at Signify to develop a customized, high-powered light source, further intensifying the system.

### 1.2 Light Source Characteristics

**Figure 2:**
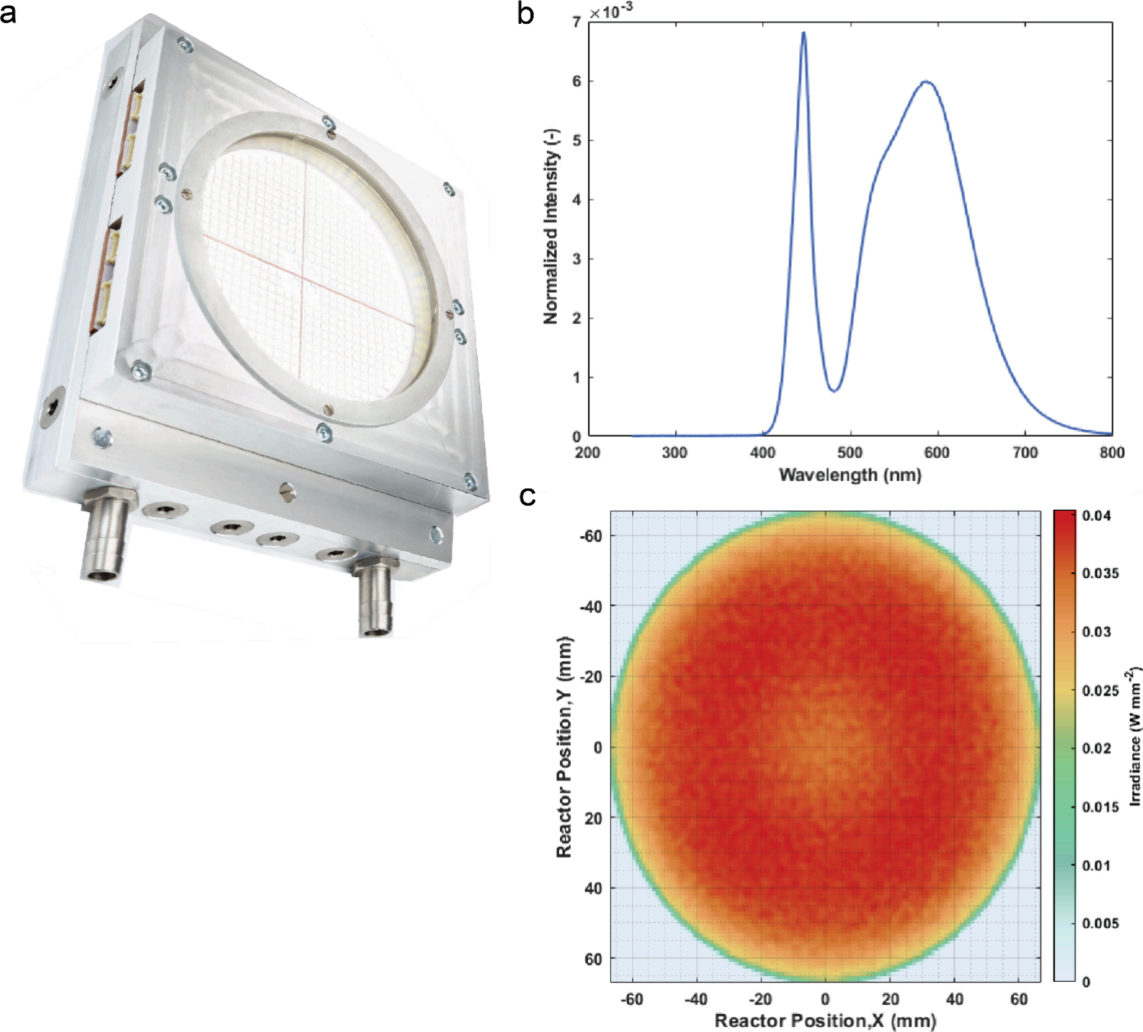
Overview of the tailored Signify light source used in this study. (a) Image of the light source depicting the cooling channel inlets and the window through which light is irradiated upon the reactor. The size of this window mimics the dimensions of the pRS-SDR. (b) Normalized spectral distribution of the light output in the range of 250–800 nm(white light of 4000K, CRI = 70). (c) Simulated light map depicting the range of light intensity over the quartz window of the pRS-SDR.

An image of the Signify light source used is shown in Figure 2a. The current design achieves a maximum optical output of 652 W over a circular area of 176.7 cm^2^. The system’s electrical input is 1750 W at peak optical output, with 1098 W dissipated as heat, necessitating water cooling for thermal management. The light source also allows variable optical output (106–652 W) by adjusting the dimming voltage. The white light source (4000K) emits across a spectrum of 250–800 nm (Figure 2b, showing the normalized spectrum at maximum output). Simulations using LightTools indicate the irradiance across the quartz window of the pRS-SDR ranges from 0.0225 and 0.0405 W mm^–2^, with fairly homogeneous irradiance across the reactor surface, as shown in Figure 2c.

### 1.3 Process Description and Schematic

**Figure 3:**
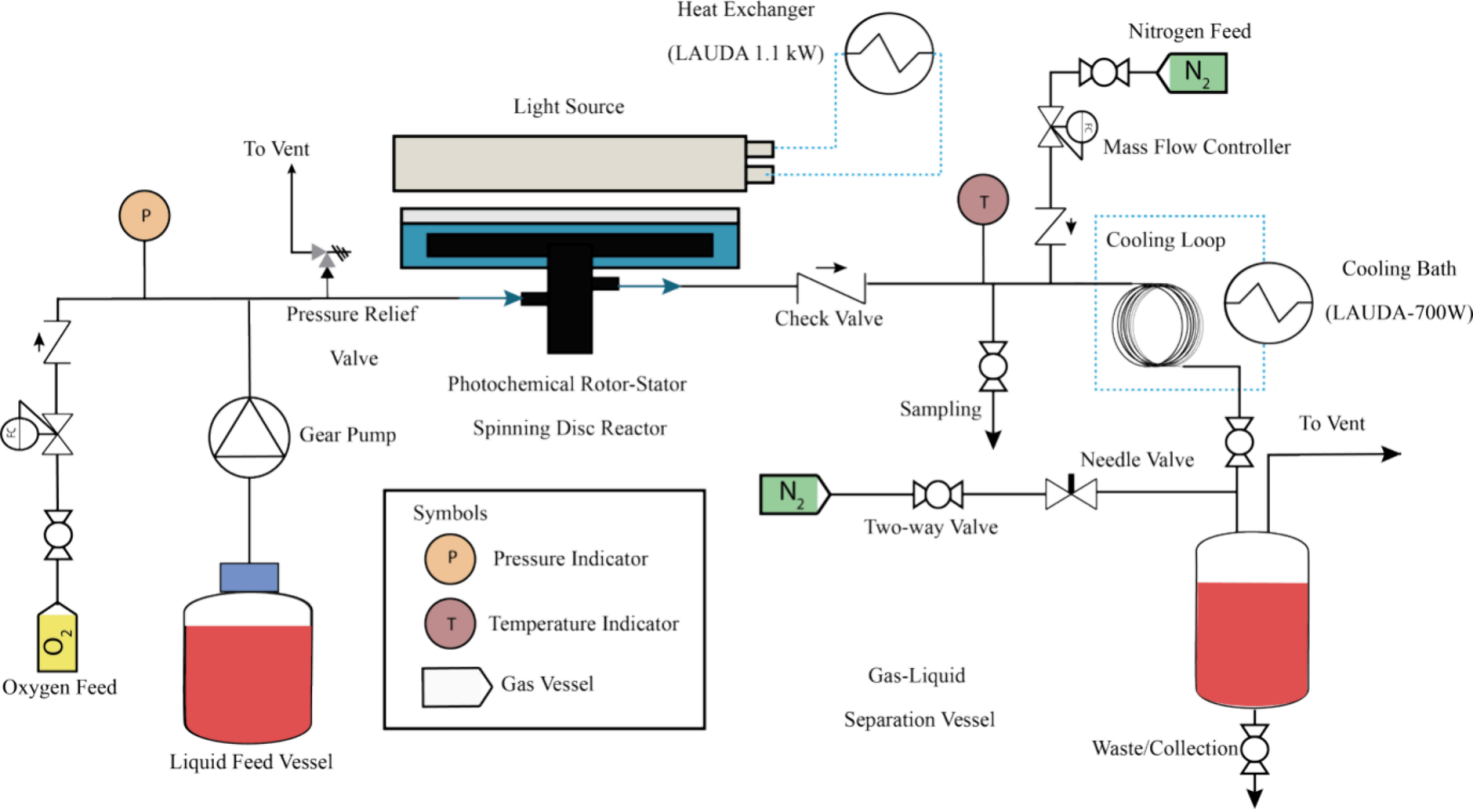
Process and instrumentation diagram of the pRS-SDR system used in this work. O_2_ was provided to the system through a gas cylinder and cofed with the liquid stream to the pRS-SDR. The outlet had a valve through which samples could be obtained while the main flow stream was passed through a cooling loop with a N_2_ feed to dilute the oxygen concentration (of any unreacted excess) in the exit stream. The exit stream was then collected in a gas–liquid separation vessel where N_2_ was continuously fed to refresh the headspace. The total liquid inventory was limited to 5 L in the whole setup during experiments.

The process schematic used in this study is shown in Figure 3. Gas (O_2_) and liquid (starting material and ethanol) streams are cofed into the reactor, and steady-state samples are collected after waiting at least six residence times. These samples are then analyzed using GC-FID to determine conversion.

Additional details on the process flow, reactor operation, and sample analysis are provided in the Methods section. It should be noted that the use of pure oxygen along with a flammable solvent such as ethanol presents a safety risk. The precautions taken to mitigate those risks are presented in the Methods section.

### 1.4 Model Reactions

**Scheme 1:**
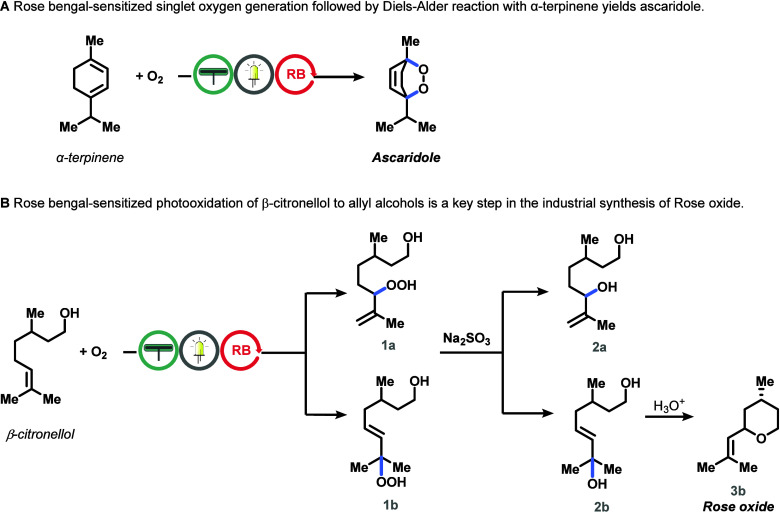
Two Model Reactions Investigated for Scale-Up in This Work (a) Reaction of α-terpinene with singlet oxygen resulting in the endoperoxide, ascaridole. (b) Reaction of β-citronellol with singlet oxygen which results in the formation of two hydroperoxides (**1a** & **1b**). Upon reduction with sodium sulfite, two different diols (**2a** & **2b**) are obtained. Rose oxide (**3b**) can be obtained after reaction with an acid. RB stands for Rose Bengal

To explore the intensification potential of the pRS-SDR with the high-powered light source, we employed two model gas–liquid photooxygenation reactions. The first was the [4 + 2] cycloaddition between α-terpinene and photochemically generated singlet oxygen to produce ascaridole, which also served as the benchmark in our previous study.^61^ The reaction of α-terpinene with singlet oxygen forms the endoperoxide ascaridole (Scheme 1a), following a [4 + 2] cycloaddition mechanism.^78^ Ascaridole is a natural compound used to treat ascariasis and as an antimalarial agent.^79^

The second was the Schenck-ene reaction between singlet oxygen and β-citronellol (Scheme 1b), which is used industrially to produce the fragrance rose oxide.^80^ The singlet oxygen reaction with β-citronellol results in the formation of two allylic hydroperoxides (moieties **1a**, **1b** in Scheme 1b). Sodium sulfite reduction produces two diols (**2a, 2b** in Scheme 1b), with diol **2b** protonating in acidic conditions to form a mixture of rose oxide stereoisomers. Only the (−)-cis isomer contributes to the rose fragrance.

Both reactions are commonly used to benchmark the performance of novel photoreactors and assess their scaling and intensification capabilities, enabling systematic comparisons with existing literature.^44,54,81−86^ In both systems, a photosensitizer (herein Rose Bengal) is employed to excite oxygen from its triplet ground state to a reactive singlet state under irradiation, which then reacts with the starting material.^79,80^ Notably, the generation of singlet oxygen is the only photon-induced step in these reactions.

A critical step in both reactions is the transport of oxygen from the gas phase to the liquid phase, where it is activated to its singlet state. If the chemical reaction rates exceed the rate of gas–liquid mass transfer, this transport step becomes rate-limiting and ultimately dictates overall productivity. In this study, by using the RS-SDR, the final productivity can be significantly enhanced due to the high gas–liquid mass transfer rates.

## 2 Results and Discussion

### 2.1 Theoretical Maximal Absorption

While the photon flux intensity on the reactor quartz window is relatively uniform (Figure 2c), the spectrum of the light source is broad due to the use of white light (Figure 2b), chosen for its ability to excite a range of visible light photocatalysts. However, for an optimized process, the light source’s emission wavelength should ideally match the photocatalyst’s absorption spectrum to maximize efficiency and improve the overall energy balance.

**Figure 4:**
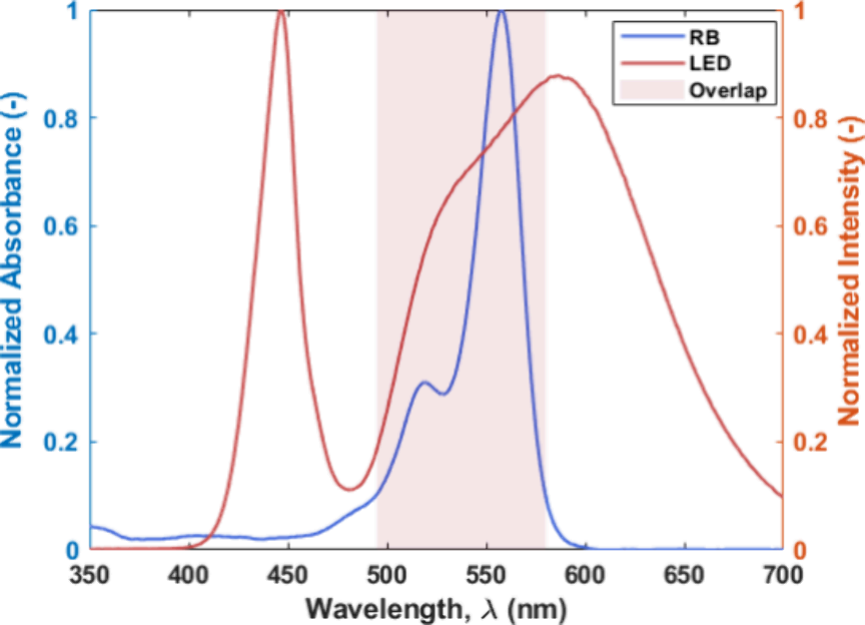
Normalized spectrum of the light source used along with the measured, normalized absorption spectrum of the Rose Bengal (RB) catalyst in ethanol between 350 and 700 nm. The figure also illustrates the overlap between the two spectra as this represents the estimated fraction of the input light intensity that can be used for the reaction.

To estimate the spectral overlap between the photocatalyst and the light source, the spectrum of Rose Bengal (1.6 × 10^–3^ M in ethanol) was measured using a UV–vis spectrometer. As shown in Figure 4, the photosensitizer absorbs only a fraction of the emitted photons. A 10% threshold based on peak absorbance was used to define the region of spectral overlap. Our analysis indicates that only about 37% of the emitted energy (in the 495–580 nm range) is useful for activating Rose Bengal, and this value was used in subsequent irradiance calculations (Table 1). Further details on these calculations are provided in the Supporting Information.

**Table 1:** Overview of the Optical Output and Relevant Optical Output at Various Dimming Conditions Used in This Study^a^

voltage (V)	optical output (W)	relevant optical output (W)	relevant irradiance (W cm ^–2^)
2.0	161	59	0.42
4.0	324	119	0.84
6.0	488	179	1.27
8.0	652	239	1.70

a The irradiance value is calculated based on the wavelength overlap of the light source and the photocatalyst spectra presented in Figure 4.

In an attempt to capture a broader range of irradiance, we tested a combination of Rose Bengal and methylene blue (which absorbs in the 500–700 nm range) as photocatalysts. However, this approach did not yield the desired results, which are discussed further in the Supporting Information.

### 2.2 Photooxidation of α-Terpinene in the pRS-SDR

**Figure 5:**
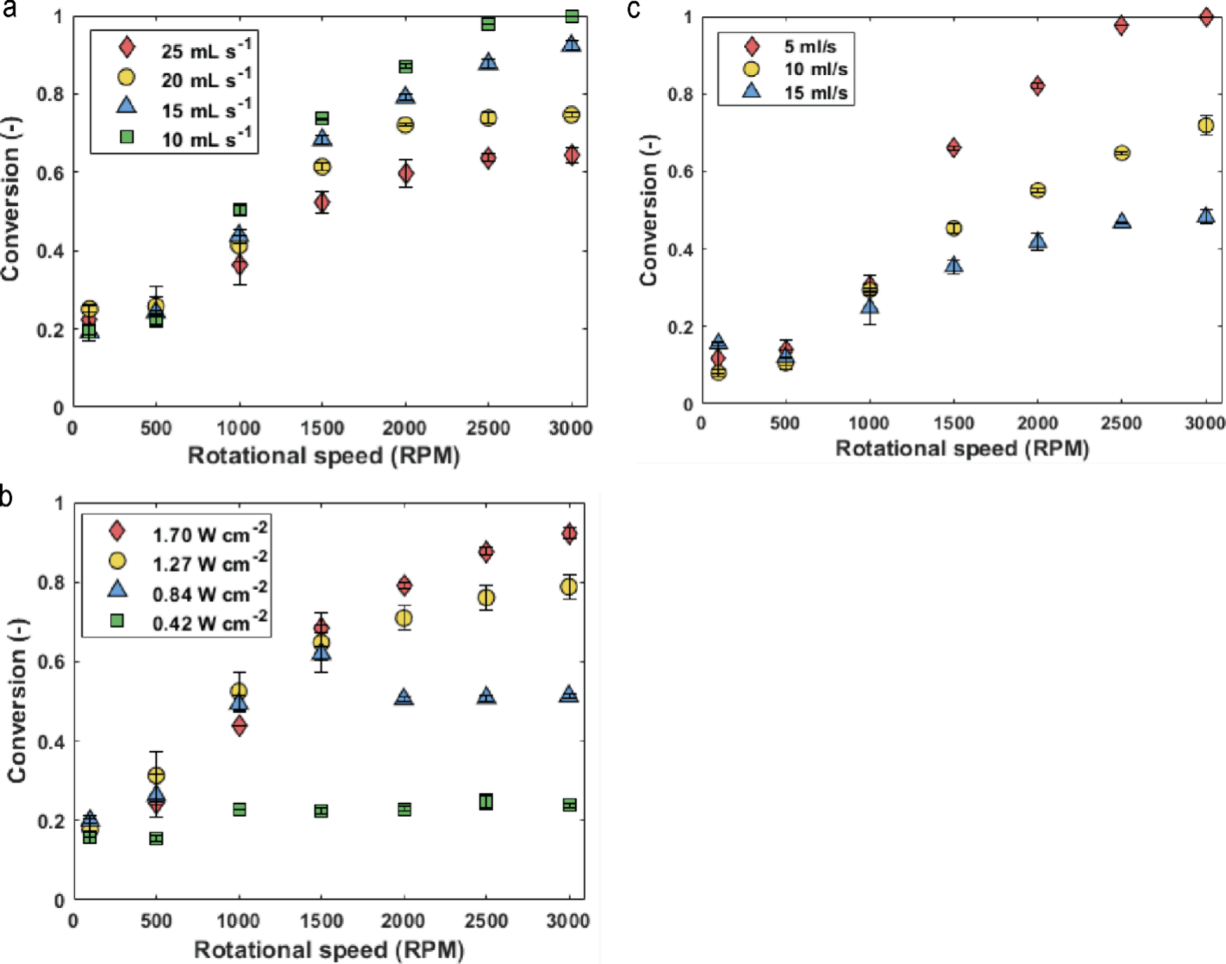
(a) Conversion of α-terpinene obtained in the pRS-SDR for various flow rates and rotation speeds at an irradiance of 1.70 W cm^–2^. The concentration of Rose Bengal in these experiments was 1 mol % while the concentration of starting material was 0.1 M, and the feed gas–liquid volumetric flow rate ratio was 3:1. (b) Conversion of α-terpinene obtained in the pRS-SDR at various irradiance values and rotation speeds at flow rate of 15 mL s^–1^. The concentration of Rose Bengal in these experiments was 1 mol %, while the concentration of starting material was 0.1 M, and the feed gas–liquid volumetric flow rate ratio was 3:1. (c) Conversion of α-terpinene obtained in the pRS-SDR at various flow rates and rotation speeds with a starting material concentration of 0.2 M. The concentration of Rose Bengal in these experiments was 1 mol % while the irradiance was 1.70 W cm ^–2^. The feed gas–liquid volumetric flow rate ratio was 3:1.

In our initial investigations, we varied the liquid flow rate to the reactor, adjusting the residence time in the pRS-SDR while operating at the highest optical intensity. The gas–liquid volumetric flow ratio was maintained at 3:1, with a Rose Bengal concentration of 1 mol % and a starting material concentration of 0.1 M, conditions chosen based on previous results obtained in the reactor.^61^ As shown in Figure 5, full conversion of α-terpinene was achieved at the highest irradiation (1.70 W cm^–2^) and 3000 rpm, with a flow rate of 10 mL s^–1^. The corresponding residence time, estimated at 2.7 s in the irradiated zone, assumes no gas holdup and represents an upper bound estimate. The strong dependence on rotation speed indicates that the reaction is mass transfer limited under high photon flux. At higher flow rates, with reduced residence time, lower conversions were observed.

Additional experiments were conducted at varying light intensities and rotational speeds with a flow rate of 15 mL s^–1^ (Figure 5b). At the lowest intensity (0.42 W cm^–2^) the system is photon-limited. At 0.84 W cm^–2^, the system initially shows mass transfer limitations, but with increasing rotation speeds—and therefore higher gas–liquid mass transfer rates—it transitions back to photon limitation. At higher intensities (1.27 W cm^–2^ and 1.70 W cm^–2^), the system enters a mixed regime where both mass transfer and irradiance influence final productivity.

Since full conversion was achieved at the highest light intensity (1.70 W cm^–2^) and a flow rate of 10 mL s^–1^, we investigated whether increasing the initial α-terpinene concentration could improve the system’s productivity and efficiency. As shown in Figure 5c, at a flow rate of 15 mL s^–1^, a conversion of 48% was observed for a starting material concentration of 0.2 M, compared to 92% for a starting material concentration of 0.1 M at the same flow rate and the same light intensity of 1.70 W cm^–2^ (Figure 5b). This suggests the system is nearing kinetic limitations and operates efficiently under the given optical conditions.

**Figure 6:**
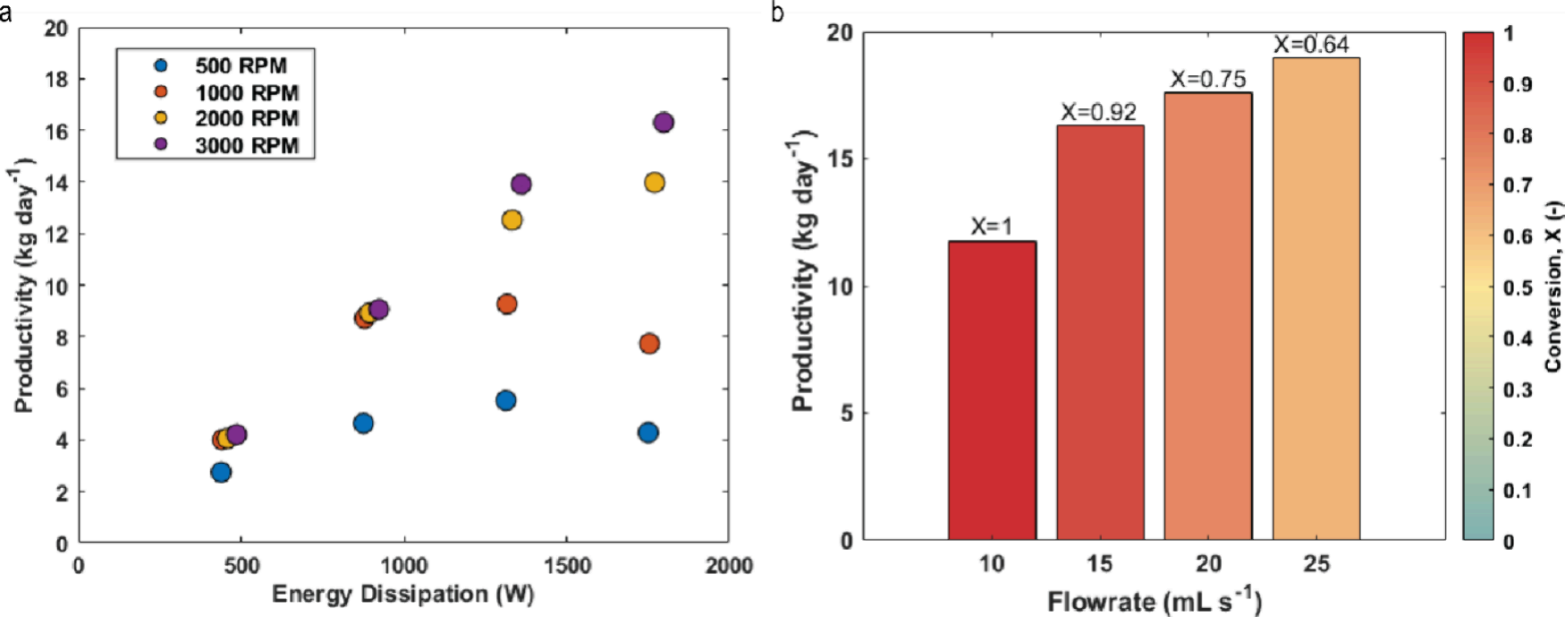
(a) Productivity (kg day^–1^) obtained for experiments conducted with 15 mL s^–1^ flow rate, 1 mol % Rose Bengal concentration, 0.1 M initial α-terpinene concentration, and a 3:1 volumetric gas–liquid flow ratio as a function of the energy dissipation (W) by the motor and the light source. The effect of rotation speed at these conditions is also indicated. (b) Varying productivity (kg day^–1^) obtained for flow rates in the range of 10–25 mL s^–1^, 1 mol % Rose Bengal concentration, 0.1 M initial α-terpinene concentration, and a 3:1 volumetric gas–liquid flow ratio at 3000 rpm. The conversions obtained for the different data points are indicated through a color map with the legend being provided on the right and the specific value being provided above the relevant bar.

We further analyzed system productivity in terms of energy dissipation (Figure 6). The electrical power input to the light source, at 1750 W for the highest optical output, had a far greater impact on energy dissipation than the motor, which dissipated only 48.5 W at 3000 rpm. Figure 6a shows that productivity (based on the obtained conversion values) is highly dependent on light intensity. The high gas–liquid mass transfer rates at elevated rotation speeds enable the reaction to operate at or near intrinsic kinetic rates under high irradiance. A maximum productivity of 19 kg day^–1^ (139 mol day^–1^) was achieved, albeit with a conversion of 64%. At higher conversion (92%), the productivity was 16.3 kg day^–1^ (120 mol day^–1^, Figure 6b). Productivity analyses for all experimental conditions are available in the Supporting Information.

Further experiments, including varying photocatalyst concentrations, pressure, gas–liquid ratios, and control experiments, are provided in the Supporting Information.

### 2.3 Photooxidation of β-Citronellol in the pRS-SDR

**Figure 7:**
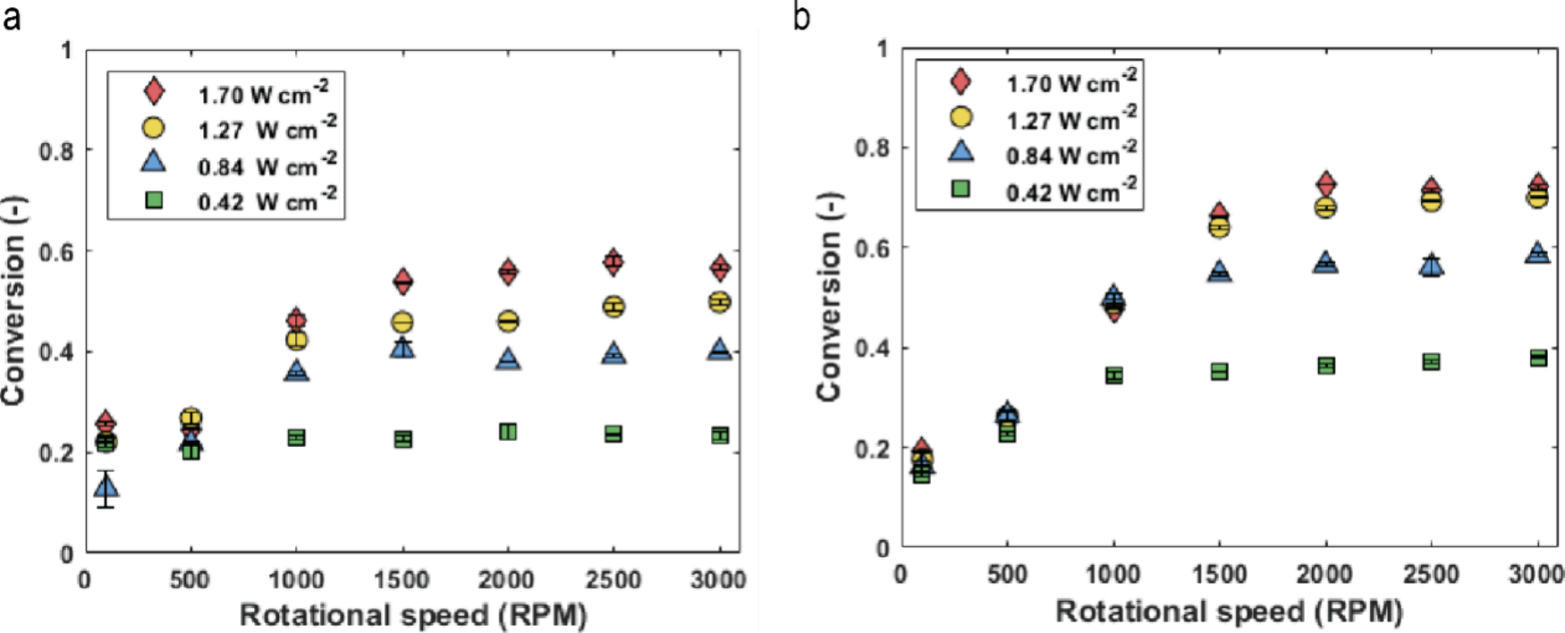
Conversion of β-citronellol obtained in the pRS-SDR at various irradiance and rotation speeds at flow rates of (a) 10 and (b) 5 mL s^–1^. The concentration of Rose Bengal in these experiments was 1 mol % while the concentration of starting material was 0.1 M. The feed gas–liquid volumetric flow rate ratio was 3:1.

We began investigating the β-citronellol photooxidation in the pRS-SDR at a liquid flow rate of 10 mL s^–1^ (gas–liquid volumetric flow ratio of 3:1, with a Rose Bengal concentration of 1 mol %), varying light intensities and rotation speeds. As shown in Figure 7a, the conversions achieved under these conditions were significantly lower than those for α-terpinene photooxidation, even at the highest light intensity of 1.70 W cm^–2^. When the flow rate was reduced to 5 mL s^–1^, increasing the residence time, conversion improved to 71% at 1.70 W cm^–2^ (Figure 7b). These results suggest that while enhanced mass transfer improves conversion, another limiting factor is at play. As Figure 7b shows, increasing light intensity beyond 1.27 W cm^–2^ does not further improve conversion. A possible explanation for the slower reaction rate is the higher energy transition state required for the “Schenk-ene” reaction compared to the Diels–Alder transition state in the ascaridole synthesis.^78^ Thus, despite using identical conditions for photosensitizer concentration, gas–liquid ratios, and starting material, the β-citronellol reaction exhibits a lower rate, even without mass or photon transport limitations for singlet oxygen generation.

**Figure 8:**
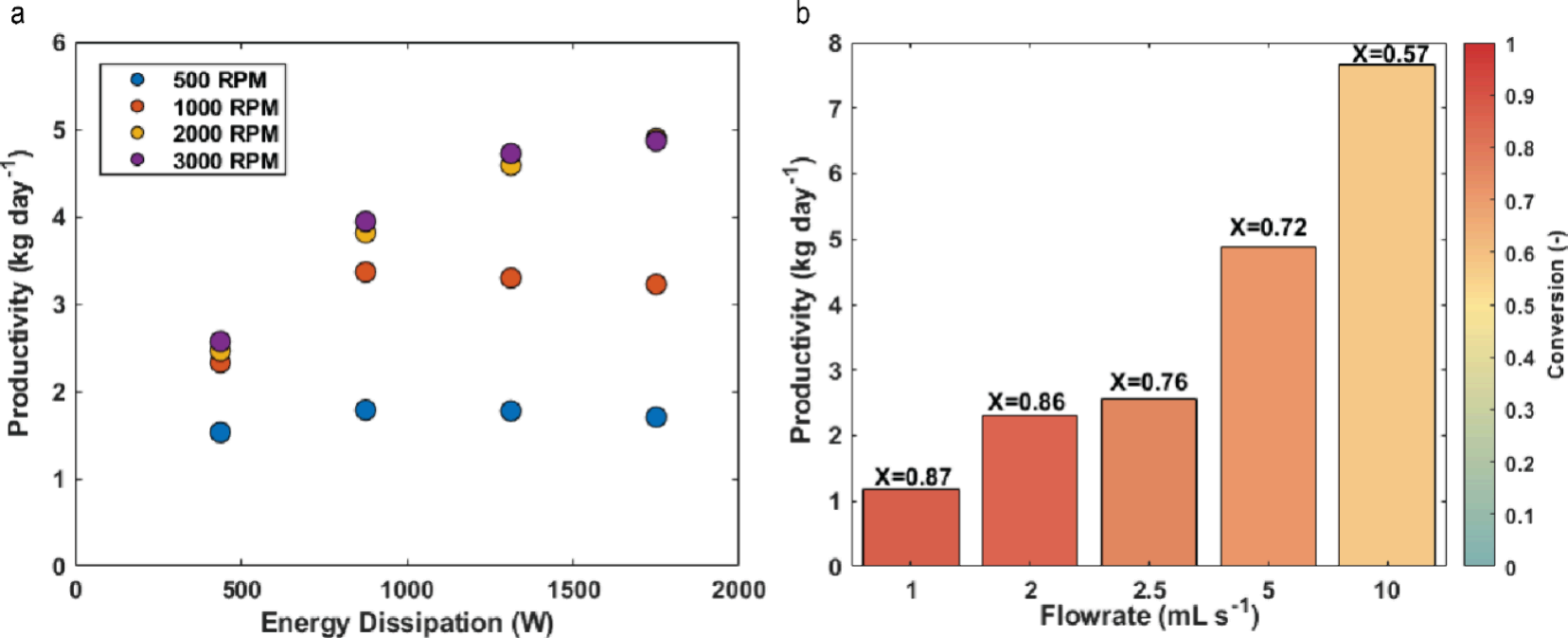
(a) Productivity (kg day^–1^) obtained for experiments conducted with 5 mL s^–1^ flow rate, 1 mol % Rose Bengal concentration, 0.1 M initial β-citronellol as a function of the energy dissipation (W) by the motor and the light source. The effect of rotation speed at these conditions is also indicated. (b) Varying productivity (kg day^–1^) obtained for flow rates in the range of 1–10 mL s^–1^, 1 mol % Rose Bengal concentration, 0.1 M initial β-citronellol concentration, and a 3:1 volumetric gas–liquid flow ratio and at 3000 rpm. The conversions obtained for the different data points are indicated through a color map with the legend being provided on the right and the specific value being provided above the relevant bar.

Due to the observed kinetic limitations in this reaction, the productivity in the pRS-SDR is lower compared to the α-terpinene reaction. As shown in Figure 8a, the energy dissipated by the light has less impact at the highest irradiance levels. The maximum productivity achieved was 9 kg day^–1^ (57.6 mol day^–1^), though at a relatively low conversion of 67%. The highest conversion obtained for this reaction was 87%, but the productivity was much lower at 1.2 kg day^–1^ (7.7 mol day^–1^, Figure 8b). Detailed productivity analyses for all experimental conditions are provided in the Supporting Information.

The photooxidation of β-citronellol is often used as a model reaction to benchmark novel photochemical reactors.^54,82^ However, as our results suggest, potential nonphotochemical kinetic limitations may cause this reaction to underpredict reactor efficacy, especially under high irradiance conditions. Further experiments involving varying photocatalyst concentrations, higher pressure, gas–liquid ratios, experiments with air as gas feed, and control tests are detailed in the Supporting Information.

### 2.4 Temperature Effects

**Figure 9:**
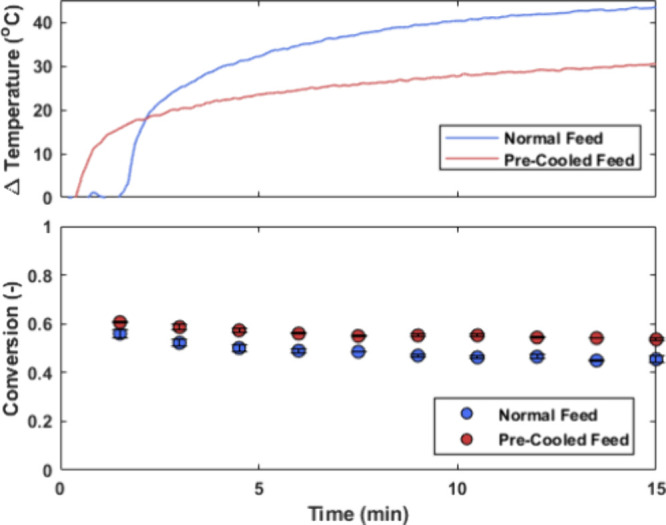
Temperature profile of the fluid measured at the outlet of the reactor for a duration of 15 min for both a precooled feed and the normal feed (room temperature). The experiments were conducted at an irradiance of 1.70 W cm^–2^, a flow rate of 5 mL s^–1^, an α-terpinene concentration of 0.2 M, and a Rose Bengal concentration of 1 mol %. The obtained conversions of α-terpinene for the corresponding experiments are also depicted. The rotation speed of the pRS-SDR was set at 1500 rpm.

The high light intensity entering the reactor leads to excess energy, not absorbed by the photocatalyst, increasing the temperature of the reaction medium. The current pRS-SDR design lacks active cooling via a heat exchanger, relying mostly on passive cooling through the reactor body. To assess the maximum temperature rise, we monitored the reaction mixture’s temperature 30 mm from the outlet using an in-line thermocouple over 15 min. This was done for a flow rate of 5 mL s^–1^ and a light intensity of 1.70 W cm^–2^ for the α-terpinene reaction, representing the most temperature-intensive conditions used in this study (longest residence time and highest light intensity). The results, shown in Figure 9, compare room temperature and precooled (5 °C) feed conditions. A temperature increase to around 55–70 °C was observed within 15 min, with the precooled feed showing a slightly lower increase (Δ*T* of 30 °C for the precooled feed versus Δ*T* of 43 °C for the normal feed). Samples taken during this period indicated only a slight negative effect on conversion, possibly due to lower oxygen solubility at higher temperatures. A similar analysis for the β-citronellol reaction (details in Supporting Information) showed no significant impact of temperature on conversion. While temperature rise is manageable for short experiments, active cooling would be necessary for large-scale production.

## 3 Conclusions and Outlook

In this work, we demonstrate the scale-up potential of the pRS-SDR for complex gas–liquid photochemical reactions using a high-power white LED light source (652 W optical output). The reactor’s high gas–liquid mass transfer rates proved essential for the photooxidation of α-terpinene, allowing the reaction to occur at or near intrinsic kinetic rates under high irradiance. The resulting productivity reached 16.3 kg day^–1^ at 92% conversion in a 27 mL irradiated reactor volume. In comparison, our previous work achieved around 1.1 kg day^–1^ primarily limited by the optical output of the used light source. These results represent a significant improvement, highlighting the pRS-SDR’s capabilities in light-driven chemistry. Interestingly, the photooxidation of β-citronellol showed much lower performance, likely due to kinetic limitations that could hinder accurate characterization of such intensified reactors. Improvements in temperature control could enhance the pRS-SDR’s appeal for scaled-up gas–liquid photochemical reactions, an aspect which will be investigated in future work.

## 4 Methods

### 4.1 pRS-SDR Process

A gear pump (Verder 2040) is used to pump the fluid from the liquid feed vessel into the pRS-SDR. An oxygen mass flow controller (Bronkhorst EL-FLOW) is used to control the oxygen flow rate from an oxygen gas bottle. The oxygen and liquid flow streams merge at a distance of 35 mm from the reactor using a Swagelok stainless steel T-piece and are cofed at the bottom of the reactor. At the distance of 25 cm from the exit of the reactor, the flow stream is split into two via a stainless steel T-piece and a stainless steel Swagelok valve is placed on one of the streams to sample the outlet of the reactor vessel. To improve the safety of the overall setup, the main outlet stream (looped coiling of 3 m, stainless steel 3/8 in. tubing) is submerged into a LAUDA bath set at 5 °C. Nitrogen is cofed into the cooling loop with a flow rate of 3 times the liquid flow rate, controlled via a mass flow controller (Bronkhorst EL-FLOW). The addition of nitrogen helps to dilute the oxygen concentration in the exit stream (of any unreacted excess) and allows for operation under safer conditions. Finally, the outlet stream is collected into a gas–liquid separation vessel where nitrogen is fed into the vessel via a needle valve to continuously refresh the gas headspace. A pressure relief valve rated at 5 bar was also placed on the pump side to ensure emergency measures were in place in the case of excess pressure build up in the reactor (rated for 6 bar). The working pressure for the majority of the experiments was 1 bar. Experiments at a higher pressure of 3 bar are presented in the Supporting Information. The total volume of the liquid feed vessel was limited to 5 L to prevent the presence of excess inventory in the reaction space. The setup was built inside an ITEM frame with continuous ventilation (75 m^3^ h^–1^ flow) inside the frame. To ensure minimum light leakage from the reactor for the optical eye safety of humans in the vicinity of the reactor, a light guard was installed, and the measured optical irradiance (blue light hazard weighted irradiance) measured on the outside of the reactor was only 2.5 W sr^–1^ m^–2^, which is 40000 times lower than the maximum allowed irradiance of 1000 W sr^–1^ m^–2^.

### 4.2 pRS-SDR Operation

Solutions of the desired concentration of either α-terpinene (TCI Chemicals, ≥ 90%) or β-citronellol (Merck, ≥ 95%) were made in ethanol (Boom Chemicals, ≥ 99.5%). The weight fraction of the photosensitizer, Rose Bengal (Thermo Fischer Scientific, ≥ 85%) was added to this solution as required. For a few experiments in this study, the use of methylene blue (TCI Chemicals, ≥ 98%) as photosensitizer was also investigated. This solution was stirred in an amber 5 L vessel to ensure homogenization of the mixture prior to experiments.

Experiments in the pRS-SDR were carried out by first switching on the LAUDA bath for the cooling loop, the heat exchanger for the light source, and the nitrogen flow to the gas–liquid separator and to the cooling loop. At this point, the gas flow to the system via the oxygen MFC was initiated to the required condition. Subsequently, the liquid flow to the system was initiated to the desired value through the liquid pump. The rotation of the pRS-SDR was then set to the desired value. At this moment, the light was switched on and the optical output was set using a knob controlling the dimming voltage. For each experimental condition, the system was operated for at least six residence times, thereby ensuring steady state, before a sample was taken at the sampling valve outlet. Two samples were taken at the sample outlet, and the error bars presented in the results represent the deviation between those two samples. The rotation speed was varied stochastically so as to not induce any hysteresis effects on subsequent data points.

To address the risks associated with using pure oxygen and a flammable solvent like ethanol, rigorous safety measures were implemented to ensure a secure experimental environment. Central to our precautions was the cooling and nitrogen dilution of the gas–liquid outlet stream, minimizing potential hazards at the source. The outlet collection vessel was maintained under a steady flow of nitrogen and directly vented to ensure safe gas dispersion. To further enhance safety, the entire setup was housed within a closed ITEM frame with continuous ventilation at a robust flow rate of 75 m^3^ h^–1^, effectively preventing the accumulation of flammable vapors. We also designed the system to limit the solvent volume within the ITEM frame to 5 L at any given time. A pressure relief valve was incorporated to automatically halt liquid flow to the reactor in emergency situations, and the mass flow controller was capped at a maximum pressure of 4 bar to prevent overpressurization. Grounding the ITEM frame, reactor frame, reactor body, and collection vessel ensured the mitigation of static charge buildup. Before initiating experiments, we conducted preliminary tests using water and water–air mixtures to quantify the reactor’s maximum temperature changes, ensuring our systems could safely handle operational extremes. The inherently short residence time and minimal reactor hold-up further reduced risks, underscoring our multifaceted approach to safety in this high-risk environment.

The samples were collected in amber glassware of 5 mL in preparation for GC-FID analysis. For the β-citronellol photooxidation reactions, the samples were treated with a molar excess of sodium sulfite (Sigma-Aldrich, ≥ 98%) to ensure the reduction of the formed peroxides to the corresponding diols. The solutions were quenched overnight before analysis. Samples for the GC-FID were prepared by taking 0.2 mL of the collected liquid and diluting it with 0.5 mL of a solution consisting of ethanol and 1 wt % of hexadecane (internal standard). In this work we analyzed the conversion of starting material. For this, a calibration curve of the starting material was obtained using GC-FID. Furthermore, for the β-citronellol photooxidation reactions, the reacted solution was reduced with sodium sulfate prior to GC analysis to convert any formed hydroperoxides to the respective diols. Samples from the α-terpinene photooxidation reaction were analyzed in a Shimadzu GC2010 Plus GC-FID with a 30 DB5 column and those from the β-citronellol reaction were analyzed in the Shimadzu, Nexis GC-2030 GC-FID with a SH-Rtx-5 Amine fused silica column. Further details on the analysis procedure can be found in the Supporting Information.

### 4.3 Light Characteristics

The output of the light source was measured in a 2-m BaSO_4_ coated integrating Ulbricht sphere (Varian Cary 17-D scanning photometer, PMT 9659QA photomultiplier tube and Keithley 2000 system electrometer). In the integrating sphere measurements, the light source (through its integrated liquid cooling system) was connected to a chiller (S&A CW-5200) and the inlet water temperature to the light source was set at 20 °C. The optical outputs of the light source were characterized at varying operating powers. A single white LED (4000K white CRI70) which was used for the light source was analyzed in a goniometer setup (LMT-GO–DS-1600) to characterize the 3-dimensional radiation profile (polar plots).

For quantifying the irradiance in the reactor, a ray-tracing model was built in LightTools using the 3D CAD files of the LED light source and the pRS-SDR, in addition to using the measured data from the integrating sphere as well as the LED radiation data from the goniometer setup. In the LightTools model, the optical properties (absorption, reflection and refraction) were defined for each component interacting with the light. The model simulations provided the resulting irradiance at the glass-reactor liquid interface of the pRS-SDR.

## Supporting Information Available

Further light source characteristics, further pRS-SDR details, control experiments in the pRS-SDR, additional data for varying photocatalyst concentrations, gas–liquid ratios, experiments at higher pressure, additional experiments at lower flow rates, experiments conducted with air as gas feed, further temperature characterizations, analytical methods, results from the mixed photocatalytic system, and relevant calculation details (PDF)
