# Supplementary material for: Scaling Up Gas–Liquid Photo-Oxidations in Flow Using Rotor-Stator Spinning Disc Reactors and a High-Intensity Light Source
Source: Org Process Res Dev. 2025 Jan 14;29(2):460–71. doi: 10.1021/acs.oprd.4c00458 (PMC11852198; doi:10.1021/acs.oprd.4c00458)
Supplement: Supplementary file 1 — op4c00458_si_001.pdf [file op4c00458_si_001.pdf]

Scaling Up Gas-Liquid Photo-oxidations in Flow Using Rotor-Stator Spinning Disc Reactors and a High-Intensity Light Source

Supporting Information

Arnab Chaudhuri[1], Wouter F.C. de Groot[1], Jasper H.A. Schuurmans[2], Stefan D.A. Zondag[2], Alessia Bianchi[1], Koen P.L. Kuijpers[3], Rémy Broersma[4], Amin Delparish[1], Matthieu Dorbec[3], John van der Schaaf[1],*, Timothy Noël[2],*

[1] Department of Chemical Engineering and Chemistry, Sustainable Process Engineering, Eindhoven University of Technology (TU/e), 5612 AZ Eindhoven, The Netherlands.

[2] Flow Chemistry Group, van't Hoff Institute for Molecular Sciences (HIMS), Universiteit van Amsterdam (UvA), 1098 XH Amsterdam, The Netherlands.

[3] Technology and Engineering Group, Janssen Research and Development, Turnhoutseweg 30, 2340 Beerse, Belgium.

[4] Signify Research, 5656 AE Eindhoven, The Netherlands

* Corresponding Author(s): j.vanderschaaf@tue.nl (John van der Schaaf)

and t.noel@uva.nl (Timothy Noël)

Supporting Information Table of Contents

1. Further Light Source Characteristics 3

1.1 Non-Normalized Light Source Radiant Flux Spectra 3

1.2 Additional Data for Variation in Radiant Flux in X/Y Position (Data from Fig 2c of main text) 3

1.3 Variation in the Optical Power Output with Dimming Voltage Applied 4

2. Further pRS-SDR Details 5

3. Additional Data in the pRS-SDR 7

3.1 *Control Experiments* 7

3.1.1 Photooxidation of α-terpinene in the RS-SDR 7

3.1.2 Photooxidation of β-citronellol in the RS-SDR 8

3.1.3 Observing for Photobleaching Effects 9

3.2 Additional Data for Photooxidation of α-terpinene in the pRS-SDR 9

3.2.1 Photocatalyst Concentration 9

3.2.2 Gas-Liquid Ratios 10

3.2.3 Higher Pressure 10

3.2.4 Productivity Analysis for Entire Data Set 11

3.3 Additional Data for Photooxidation of β-citronellol in the RS-SDR 11

3.3.1 Photocatalyst Concentration 11

3.3.2 Gas-Liquid Ratios 12

3.3.3. Starting Material Concentration 12

3.3.4 Data for Flowrate of 2.5 mL s^{-1} 13

3.3.5 Data for Flowrate of 2 mL s^{-1s} 13

3.3.6 Data for Flowrate of 1 mL s^{-1} 14

3.3.7 Productivity Analysis for Entire Data Set 14

3.3.8 Temperature Experiments 15

3.3.9 Experiments With Air As Gas Feed 15

4. Further Temperature Characterization 16

5. Analytical Methods 17

6. Mixed Photocatalyst System 20

7. Calculation Details 21

7.1 Relevant Optical Output 21

7.2 Optical Flux 21

7.4 Calculation of Productivity 21

7.5 Calculation of Energy Dissipation Rate 21

1. Further Light Source Characteristics

1.1 Non-Normalized Light Source Radiant Flux Spectra

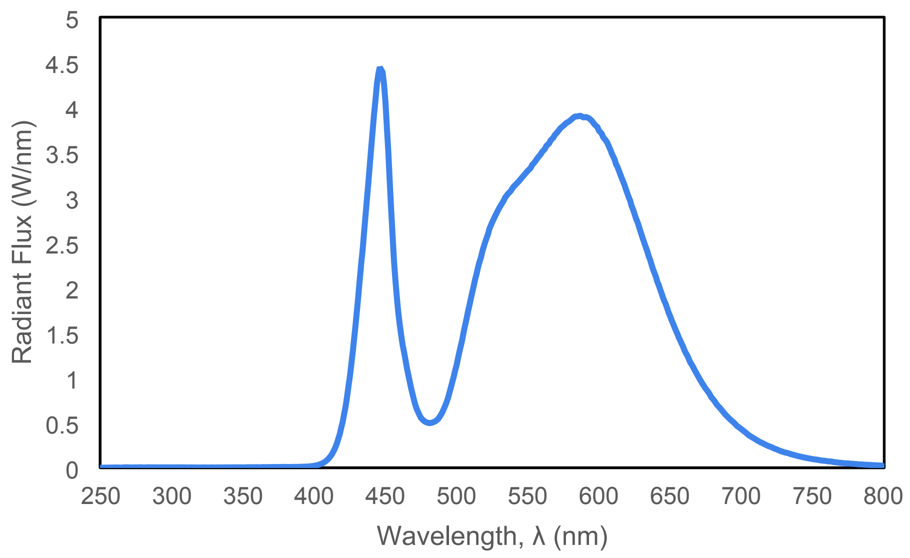

SI Figure 1: The non-normalized radiant flux spectra of the light source at the highest irradiance condition.

1.2 Additional Data for Variation in Radiant Flux in X/Y Position (Data from Fig 2c of main text)

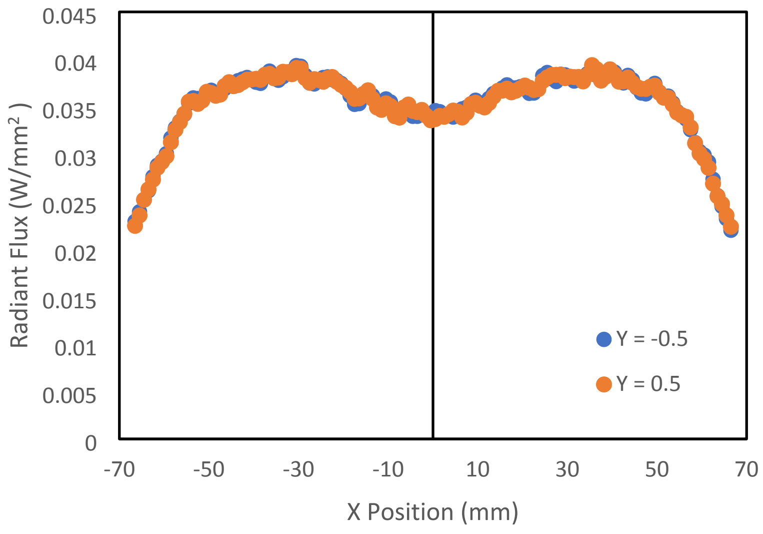

SI Figure 2: The variation of radiant flux in the x-coordinate at the Y=0.5 mm and Y=-0.5 mm coordinate positions.

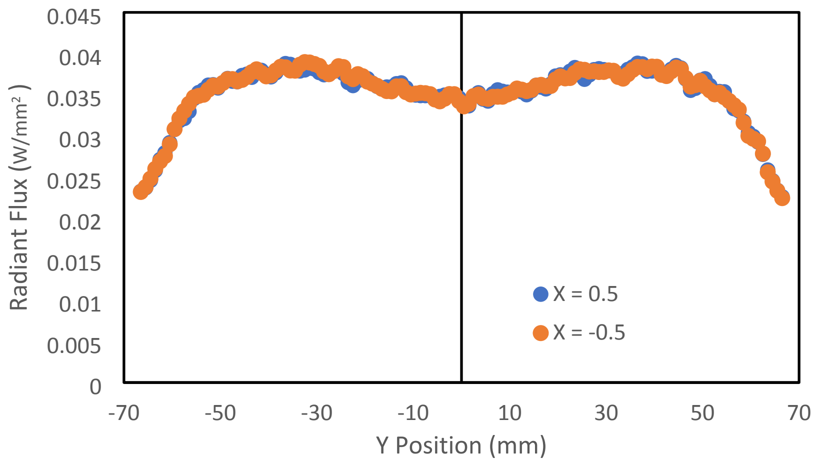

SI Figure 3: The variation of radiant flux in the y-coordinate at the X=0.5 mm and X=-0.5 mm coordinate positions.

1.3 Variation in the Optical Power Output with Dimming Voltage Applied

Signify provided a Voltage vs Optical Power Output (W) curve (graph below), from which we were able to make a calibration line and interpolate to the optical power output values used in the study (SI Table 1).

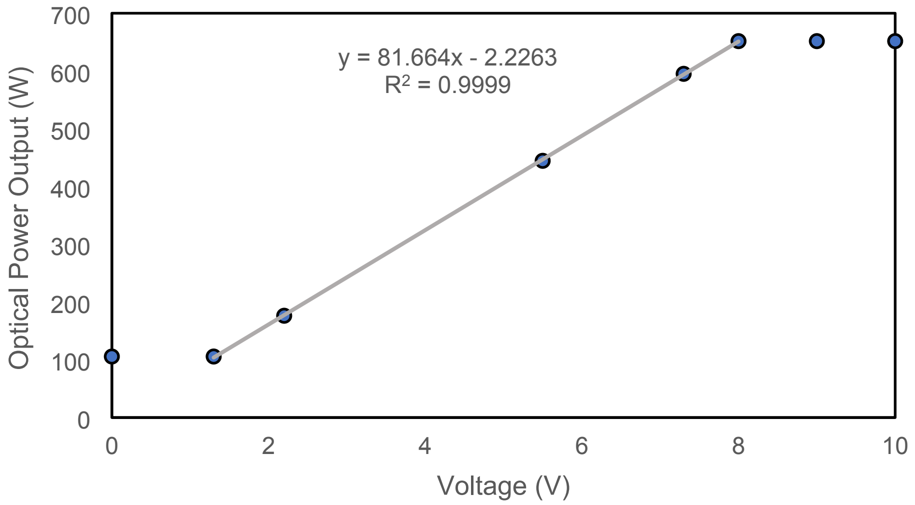

SI Figure 4: The calibration curve used to obtain the optical power output values at the dimming voltages used.

SI Table 1: An overview of the optical output and relevant optical output at various dimming conditions used in this study. The irradiance value is calculated based on the wavelength overlap of the light source and the photocatalyst spectra.

Voltage (V)	Optical Output (W)	Relevant Optical Output (W)	Irradiance (W cm $^{-2}$)
1.0	106	29	0.21
2.0	161	59	0.42
3.0	243	89	0.63
4.0	324	119	0.84
5.0	406	149	1.06
6.0	488	179	1.27
7.0	569	209	1.48
8.0	652	239	1.70

2. Further pRS-SDR Details

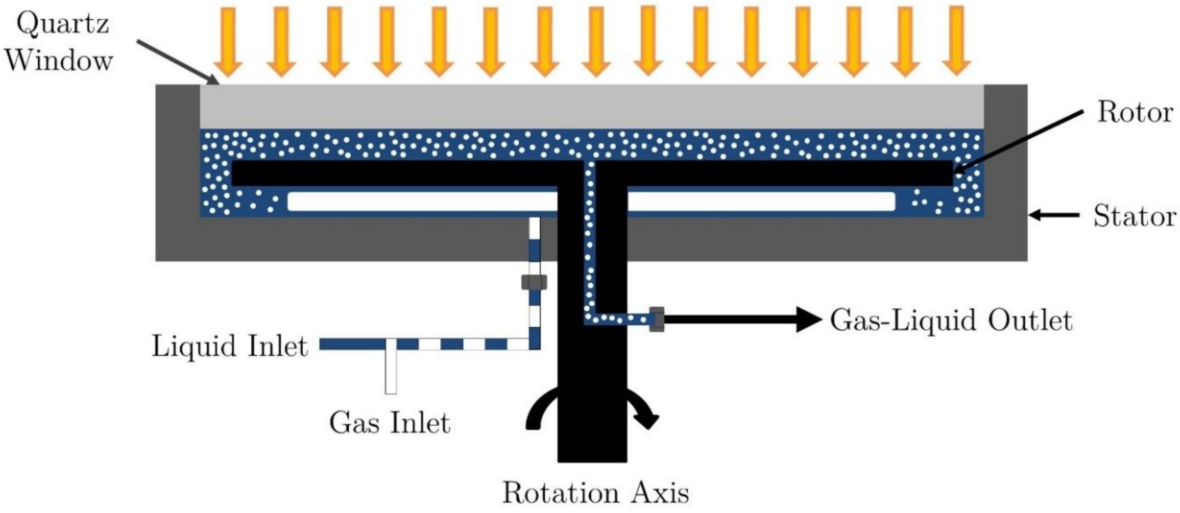

SI Figure 5: The image above illustrates the most probable type of gas-liquid flow behaviour in the pRS-SDR, where a dispersed gas-liquid phase is formed on the top side, the volume which is also irradiated.

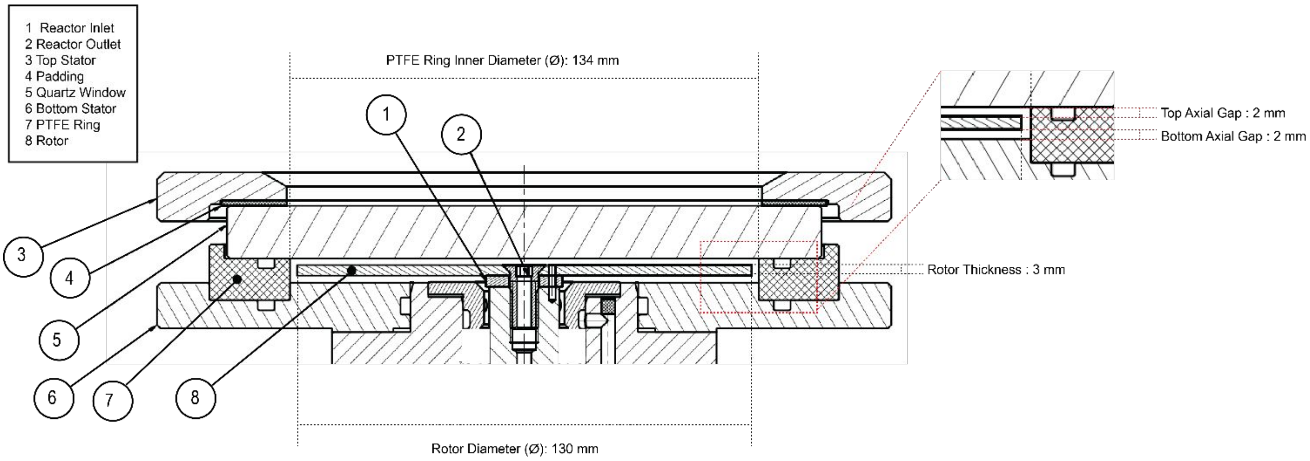

SI Figure 6: Further details regarding the dimensions of the rotor, stator and rotor-stator gap distances for the pRS-SDR used in this study. The quartz window has a thickness of 15 mm and the material used is GE-124 fused quartz.

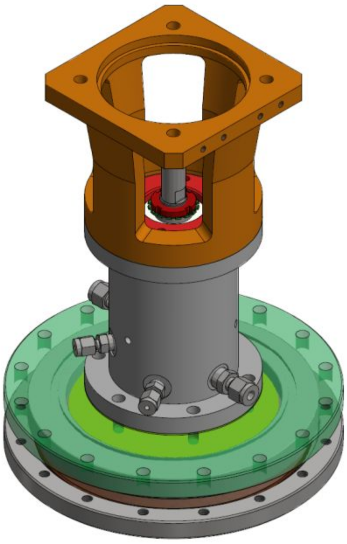

SI Figure 7: A 3D model of the pRS-SDR illustrating the reactor inlet/outlets as well as seal flushing ports.

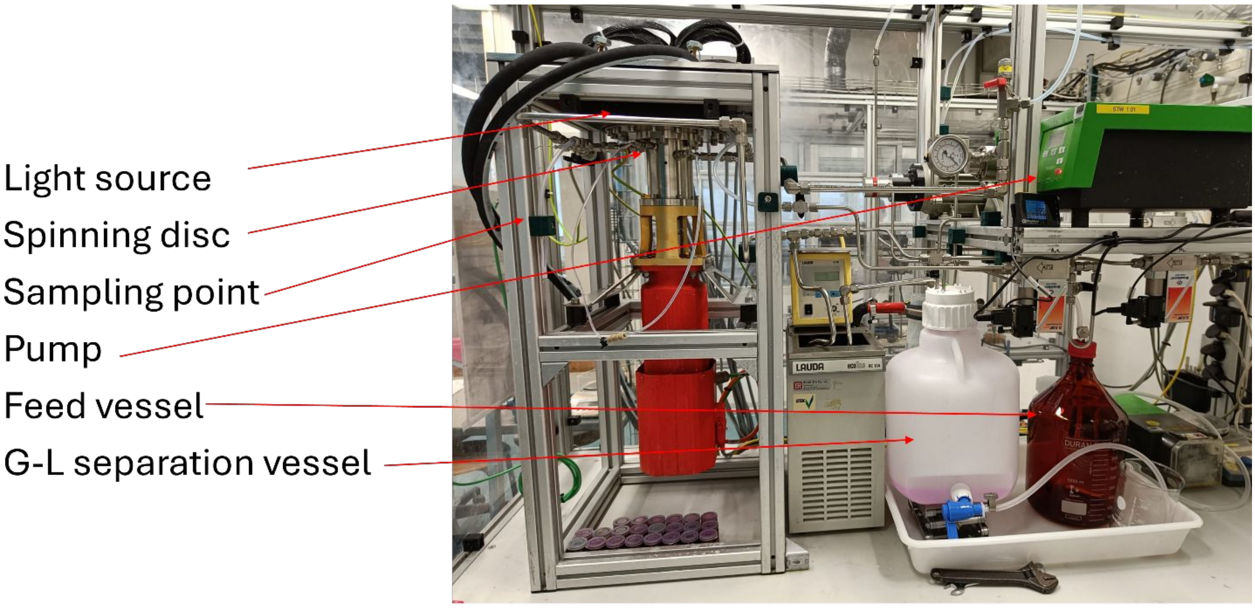

SI Figure 8: The image of the actual setup built inside the ITEM frame with various components labelled.

3. Additional Data in the pRS-SDR

3.1 *Control Experiments*

3.1.1 Photooxidation of α-terpinene in the RS-SDR

3.1.1.1 Control Experiments without Light

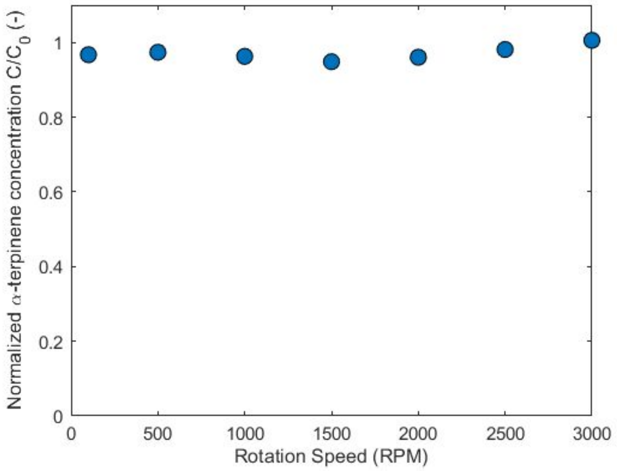

SI Figure 9:No light, flowrate of 15 mL s^{-1}, 3:1 gas-liquid ratio and Rose Bengal concentration of 1 mol % and α-terpinene concentration of 0.1 M.

3.1.1.2 Control Experiments without Photocatalyst

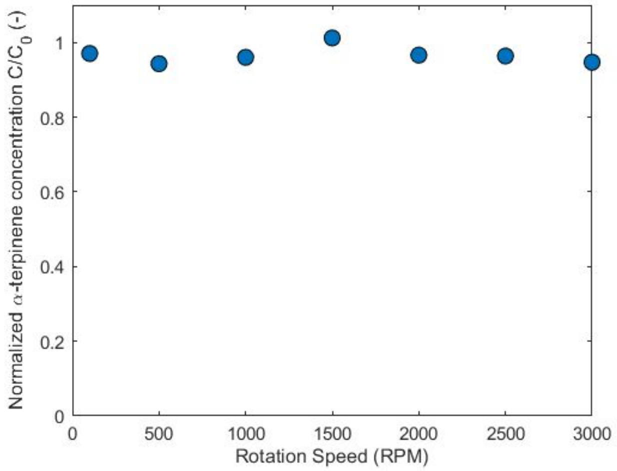

SI Figure 10: No photocatalyst, Light Irradiance at 1.70 W cm^{-2}, flowrate of 15 mL s^{-1}, 3:1 gas-liquid ratio and α-terpinene concentration of 0.1 M.

3.1.2 Photooxidation of β-citronellol in the RS-SDR

3.1.2.1 Control Experiments without Light

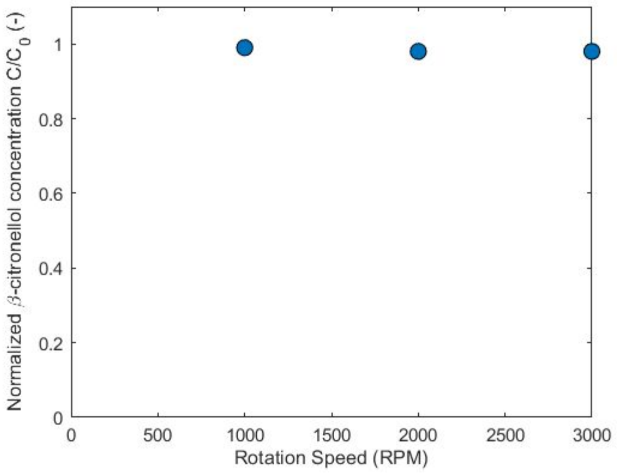

SI Figure 11:No light, Rose Bengal concentration of 1 mol %, flowrate of 5 mL s^{-1}, gas-liquid ratio of 3:1, β-citronellol concentration of 0.1 M .

3.1.2.2 Control Experiments without Photocatalyst

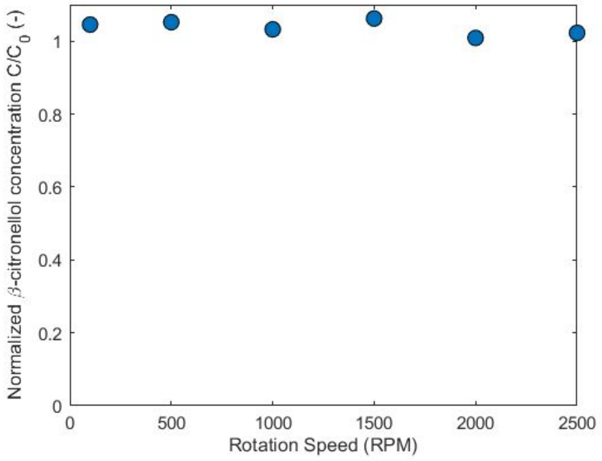

SI Figure 12: No photocatalyst, Light Irradiance at 1.70 W cm^{-2}, flowrate of 5 mL s^{-1} , gas-liquid ratio of 3:1, β-citronellol concentration of 0.1 M .

3.1.3 Observing for Photobleaching Effects

To ensure that significant photobleaching of the photocatalyst was not being observed during the reaction, a few experiments were conducted using 1 mol % Rose Bengal solution with oxygen flow in a 3:1 gas-liquid volumetric flow ratio. As the results in the Figure below illustrates, for the conditions tested, the Rose Bengal concentration (measured using an offline UV-vis) remained relatively constant. Therefore, this led us to conclude that due to the short duration of time that the photocatalyst spent in the pRS-SDR photobleaching could be avoided. The samples were obtained after waiting at least 6 residence times for steady state.

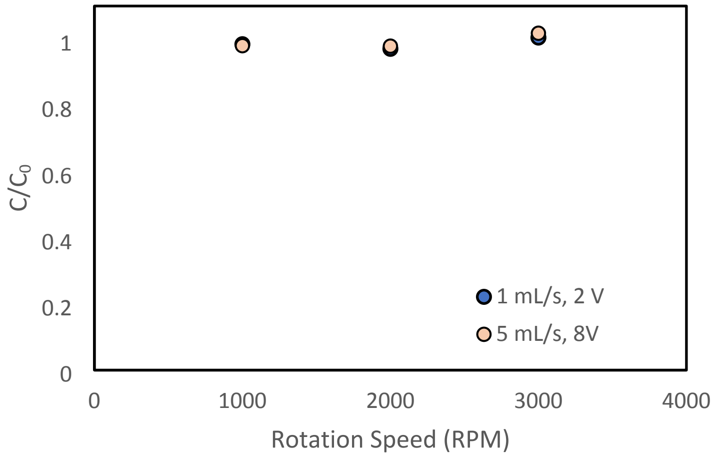

SI Figure 13: The normalized rose Bengal concentration measured at the outlet of the reactor for flow rates of 1 mL s^{-1} and 5 mL s^{-1} at 2 V (Irradiance of 0.42 W cm^{-2}) and 8 V (Irradiance of 1.70 W cm^{-2}) of light respectively and for various rotation speeds. The oxygen flow rate was kept at the 3:1 gas-liquid volumetric ratio, the most often used conditions in the main experiments of this study.

3.2 Additional Data for Photooxidation of α-terpinene in the pRS-SDR

3.2.1 Photocatalyst Concentration

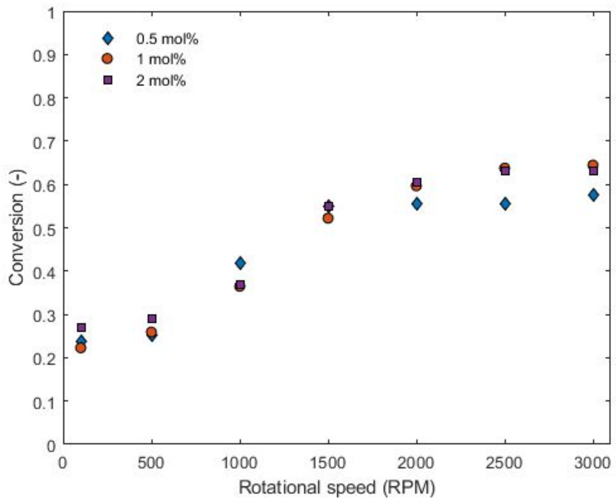

SI Figure 14: The effect of varying Rose Bengal concentrations on conversion tested in the pRS-SDR with an irradiance of 1.70 W cm^{-2}, a flowrate of 25 mL s^{-1}, an initial α-terpinene concentration of 0.1 M. The gas-liquid ratio was 3:1.

3.2.2 Gas-Liquid Ratios

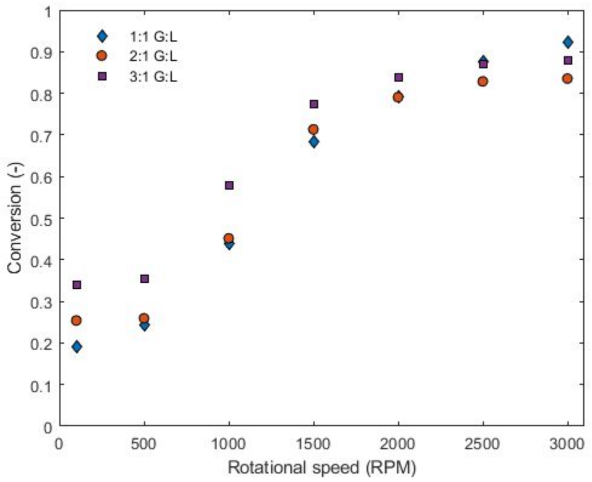

SI Figure 15: The effect of varying gas-liquid flow ratios on conversion in the pRS-SDR. The Rose Bengal concentration was 1 mol %, an irradiance of 1.70 W cm^{-2}, a flowrate of 15 mL s^{-1}, an initial α-terpinene concentration of 0.1 M

3.2.3 Higher Pressure

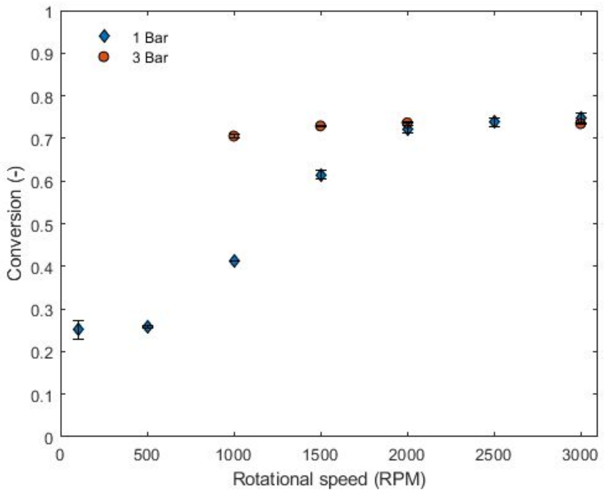

SI Figure 16: The effect of higher pressure on the conversion of α-terpinene in the pRS-SDR. The Rose Bengal concentration was 1 mol %, an irradiance of 1.70 W cm^{-2}, a flowrate of 20 mL s^{-1}, an initial α-terpinene concentration of 0.1 M. The gas-liquid flow ratio was 3:1.

3.2.4 Productivity Analysis for Entire Data Set

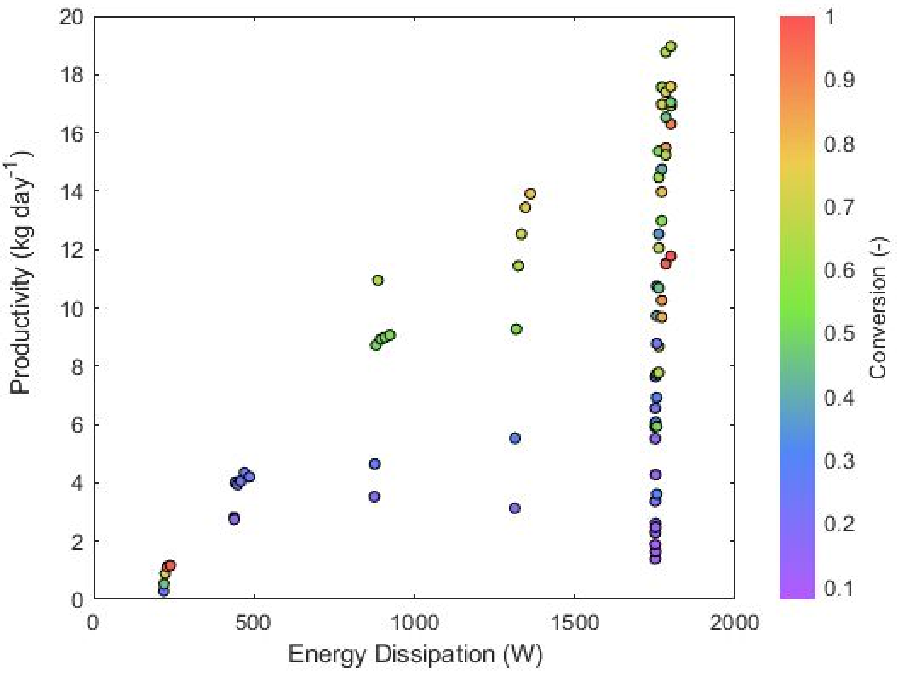

SI Figure 17: The range in productivity obtained in comparison to the energy dissipated (light and pRS-SDR) for the entire set of experiments conducted with α-terpinene. The color map provides a visual representation of the conversion obtained at the particular productivity and energy dissipation value.

3.3 Additional Data for Photooxidation of β-citronellol in the RS-SDR

3.3.1 Photocatalyst Concentration

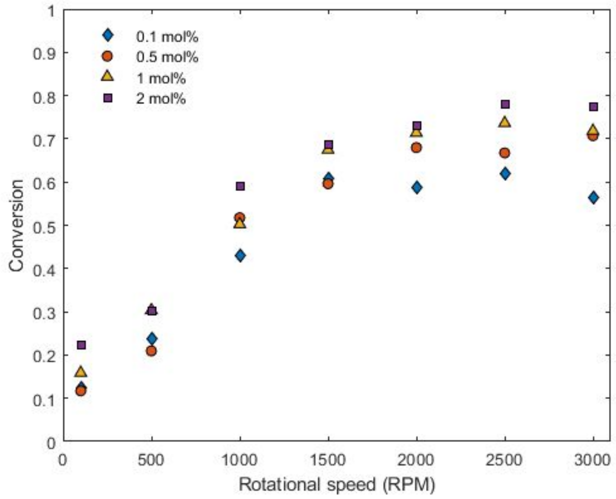

SI Figure 18: The effect of varying Rose Bengal concentrations on conversion tested in the pRS-SDR with an irradiance of 1.70 W cm^{-2}, a flowrate of 5 mL s^{-1}, an initial β-citronellol concentration of 0.1 M. The gas-liquid ratio was 3:1.

3.3.2 Gas-Liquid Ratios

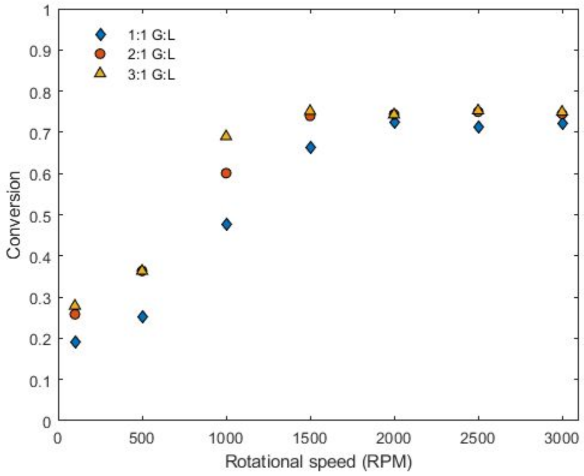

SI Figure 19: The effect of varying gas-liquid flow ratios on conversion in the pRS-SDR. The Rose Bengal concentration was 1 mol %, an irradiance of 1.70 W cm^{-2}, a flowrate of 5 mL s^{-1}, an initial β-citronellol concentration of 0.1 M

3.3.3. Starting Material Concentration

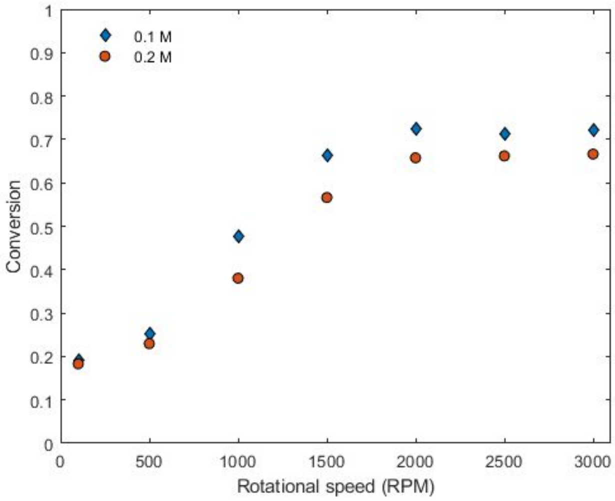

SI Figure 20: The effect of varying initial starting material concentration of β-citronellol on conversion in the pRS-SDR. The Rose Bengal concentration was 1 mol %, an irradiance of 1.70 W cm^{-2}, a flowrate of 5 mL s^{-1}. The gas-liquid flow ratio was 3:1.

3.3.4 Data for Flowrate of 2.5 mL s^{-1}

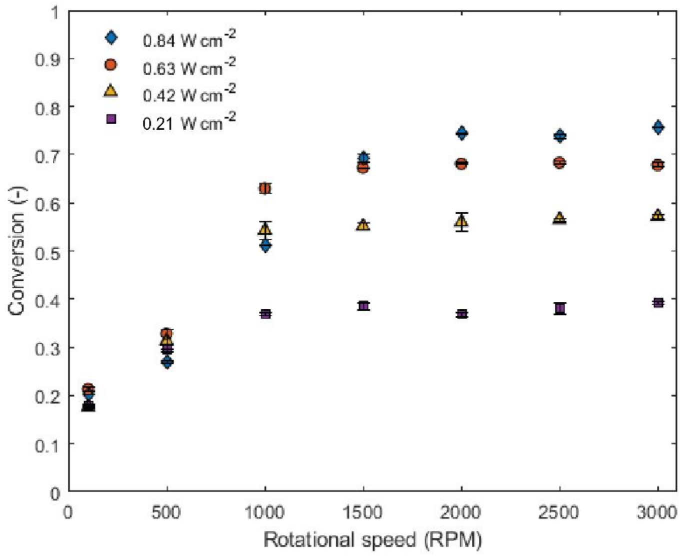

SI Figure 21: The conversion of β-citronellol obtained in the pRS-SDR at various irradiance and rotation speeds at flowrates of 2.5 mL s^{-1}. The concentration of Rose Bengal in these experiments was 1 mol % while the concentration of starting material was 0.1 M. The feed gas-liquid volumetric flowrate ratio was 3:1.

3.3.5 Data for Flowrate of 2 mL s^{-1s}

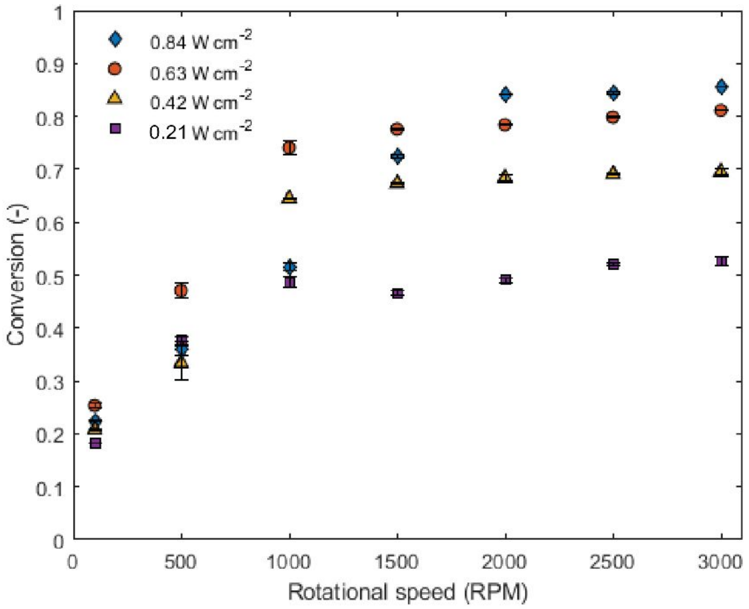

SI Figure 22: The conversion of β-citronellol obtained in the pRS-SDR at various irradiance and rotation speeds at flowrates of 2 mL s^{-1}. The concentration of Rose Bengal in these experiments was 1 mol % while the concentration of starting material was 0.1 M. The feed gas-liquid volumetric flowrate ratio was 3:1.

3.3.6 Data for Flowrate of 1 mL s^{-1}

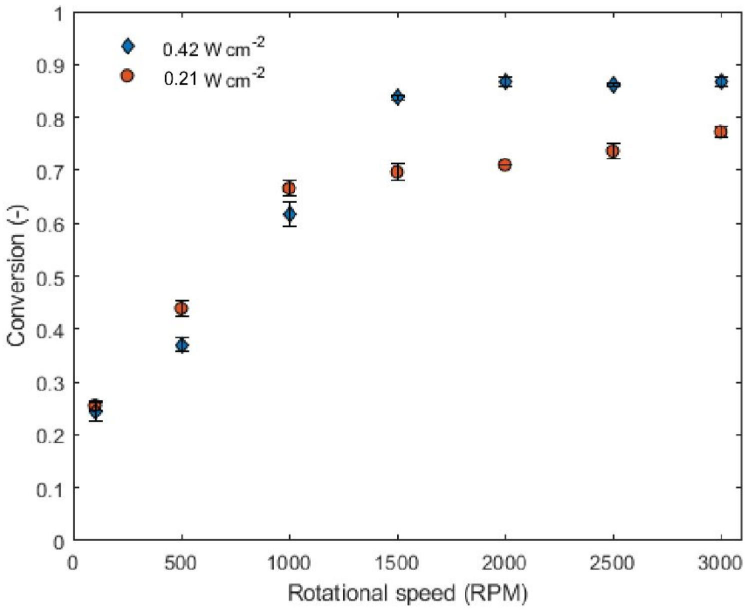

SI Figure 23: The conversion of β-citronellol obtained in the pRS-SDR at various irradiance and rotation speeds at flowrates of 1 mL s^{-1}. The concentration of Rose Bengal in these experiments was 1 mol % while the concentration of starting material was 0.1 M. The feed gas-liquid volumetric flowrate ratio was 3:1.

3.3.7 Productivity Analysis for Entire Data Set

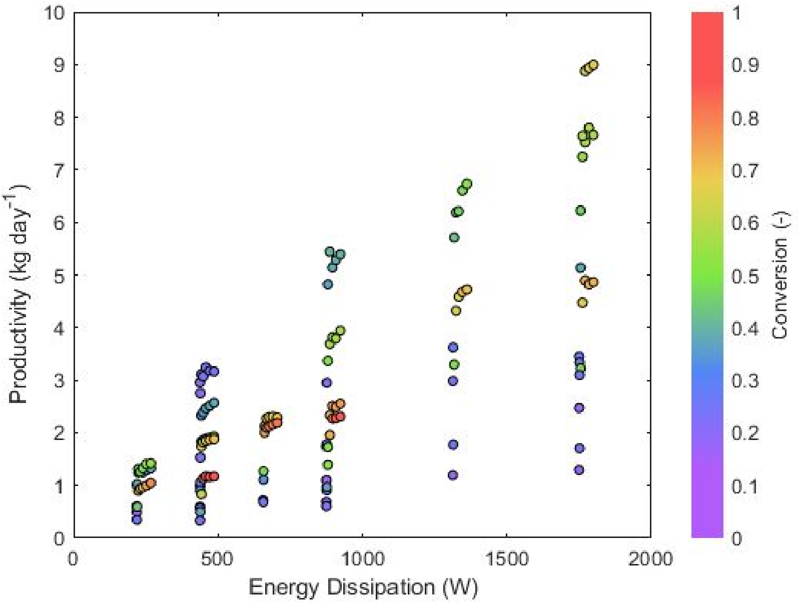

SI Figure 24: The range in productivity obtained in comparison to the energy dissipated (light and pRS-SDR) for the entire set of experiments conducted with β-citronellol. The color map provides a visual representation of the conversion obtained at the particular productivity and energy dissipation value.

3.3.8 Temperature Experiments

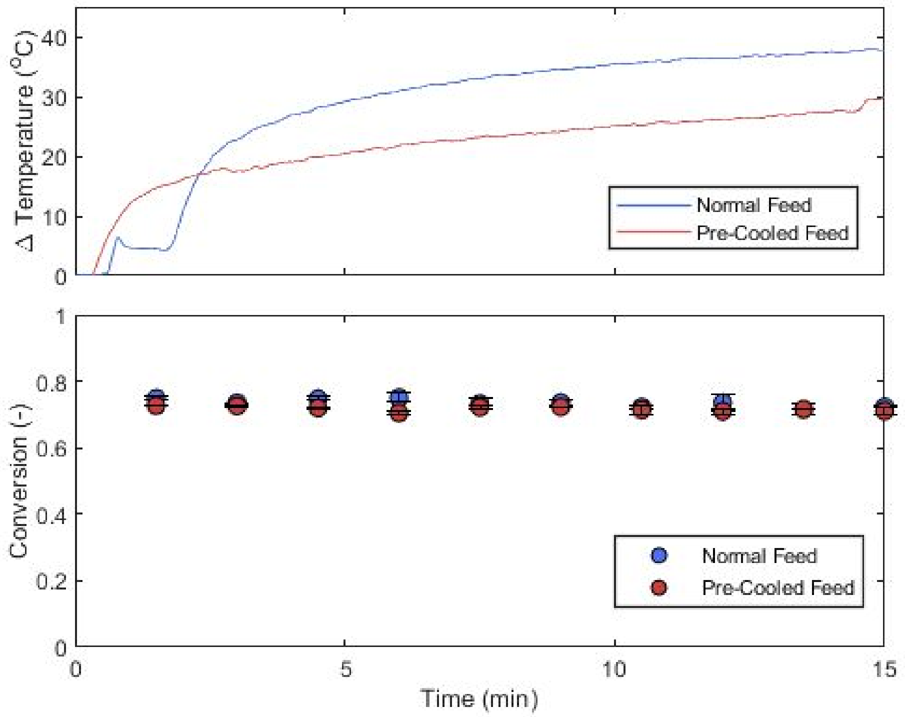

SI Figure 25: The temperature profile of the fluid measured at the outlet of the reactor for a duration of 15 minutes for both a pre-cooled feed and the normal feed (room temperature). The experiments were conducted at an irradiance of 1.70 W cm^{-2}, a flowrate of 5 mL s^{-1}, a β-citronellol concentration of 0.1 M and a Rose Bengal concentration of 1 mol %. The obtained conversions of α-terpinene for the corresponding experiments are also depicted. The rotation speed of the pRS-SDR was set at 1500 RPM.

3.3.9 Experiments With Air As Gas Feed

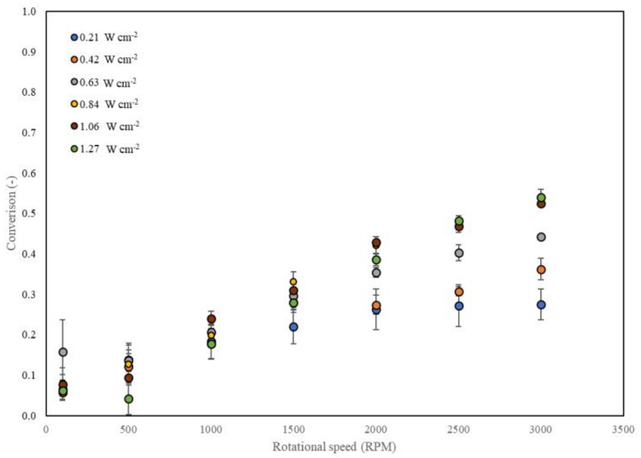

SI Figure 26: The conversion of β-citronellol obtained in the pRS-SDR with air as gas feed at various irradiance and rotation speeds at flowrates of 5 mL s^{-1}. The concentration of Rose Bengal in these experiments was 1 mol % while the concentration of starting material was 0.1 M. The feed gas-liquid volumetric flowrate ratio was 3:1.

4. Further Temperature Characterization

To determine the range of temperature rise in the reactor and operate under safe conditions, , characterization for the temperature increase with varying light power input was carried before starting the photooxidation experiments. The experiments were conducted for 30 residence times (based on the flowrate used, and considering an irradiated volume of 27 mL) with the temperature being logged via the use of an in-line thermocouple. The solvent used for these experiments was ethanol. The figure below shows the final temperature obtained for the various experimental conditions. These tests allowed us to set the boundaries for safely operating the reactor without having excessive temperature increase.

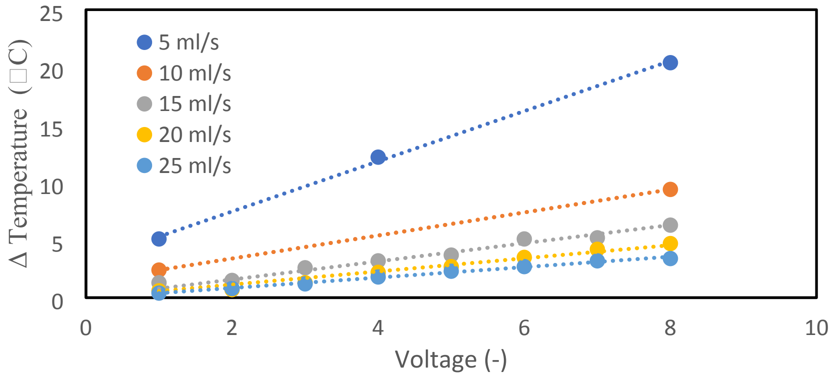

SI Figure 27: Experiments for the increase in temperature at the outlet of the reactor for various voltages and flowrates at a rotation of 1500 RPM in the pRS-SDR. No gas was used in this experiment. The temperature reported here is the increase observed after waiting 30 residence times.

5. Analytical Methods

GC-FID analysis was carried out as specified in the main text. An example of the GC spectrum of α-terpinene can be seen below, along with the internal standard peak. To fully convert the starting material, a batch setup detailed in our previous work[1], was used. The GC spectrum at full conversion has also been illustrated in the figure below.

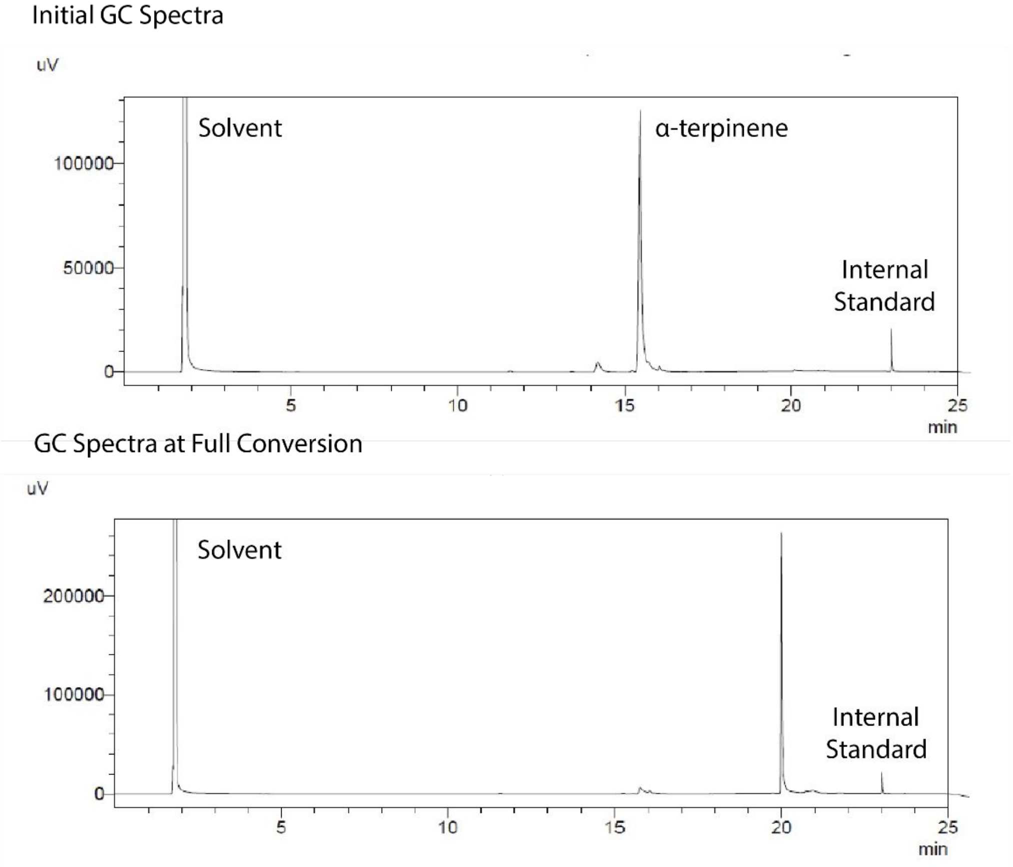

SI Figure 28: The GC spectra of initial α-terpinene and at full conversion of the starting material. The peak for the internal standard can also be seen in the spectrum above.

A calibration curve (shown in the Figure below) was made to obtain the concentration at the outlet of the pRS-SDR, from which the conversion of starting material could be obtained. This calibration curve was made by making solutions of varying mass ratios of internal standard and α-terpinene.

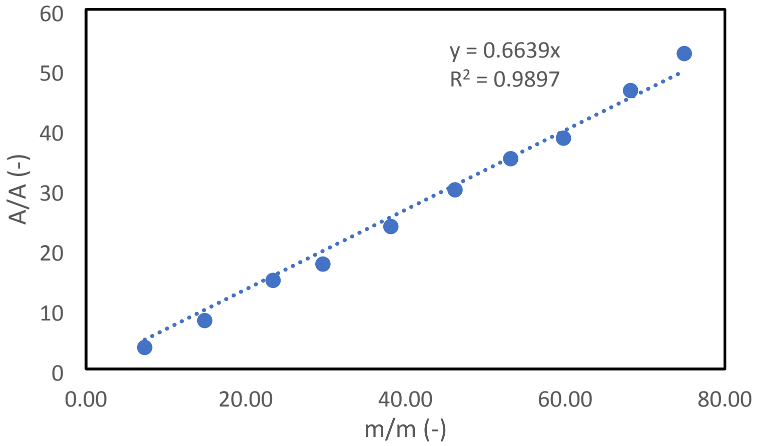

SI Figure 29: The GC calibration curve of α-terpinene. The y-axis represents the area ratios of the α-terpinene and internal standard from the GC-FID analysis of the calibration samples while the x-axis represents the mass ratios of the α-terpinene and internal standard from the preparation of the calibration samples.

A similar procedure was followed for the case of β-citronellol and the relevant graphs (GC spectrum and calibration curve) can be found below.

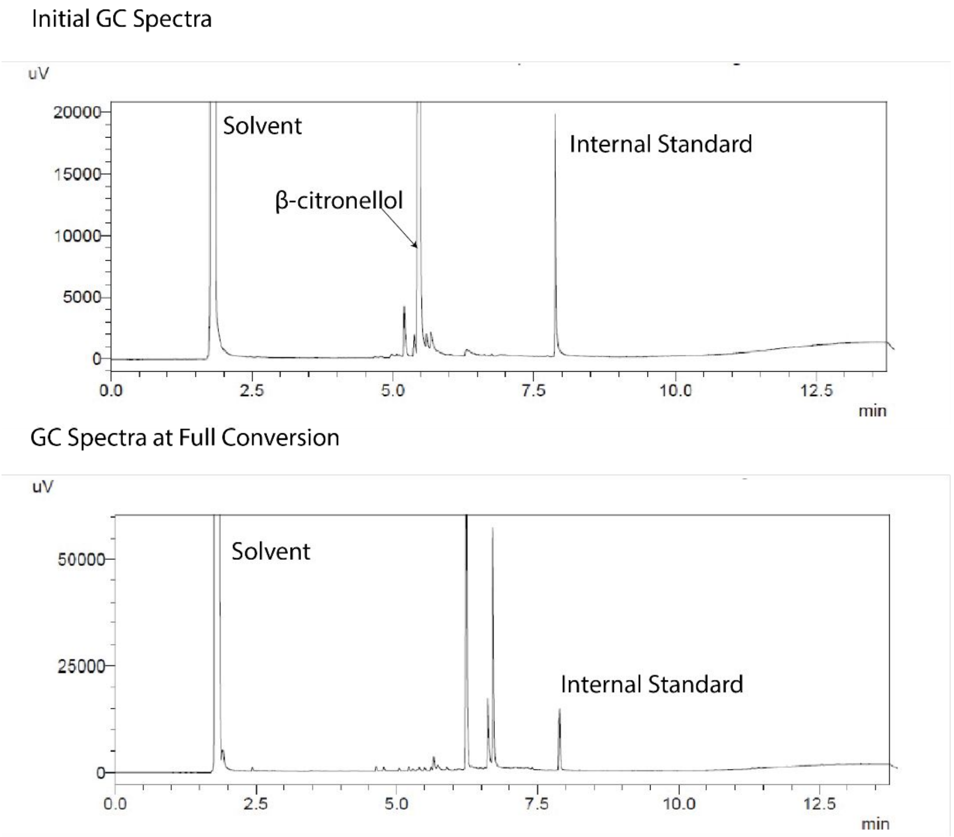

SI Figure 30: The GC spectra of initial β-citronellol and at full conversion of the starting material. The peak for the internal standard can also be seen in the spectrum above.

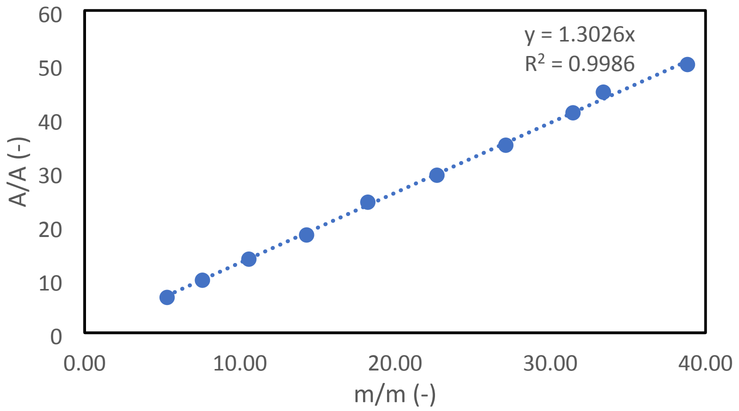

SI Figure 31: The GC calibration curve of β-citronellol. The y-axis represents the area ratios of the β-citronellol and internal standard from the GC-FID analysis of the calibration samples while the x-axis represents the mass ratios of the β-citronellol and internal standard from the preparation of the calibration samples.

6. Mixed Photocatalyst System

The results obtained for the mixed photocatalyst system (RB+MB) is illustrated below. As can be observed, the use of the combined system did not lead to improved performance in comparison to the use of only RB photocatalyst. In fact, slightly worse conversions for α-terpinene were obtained. While it is not entirely clear why this was the case, it could be due to the interaction between the activated state of the photocatalysts causing faster deactivation.

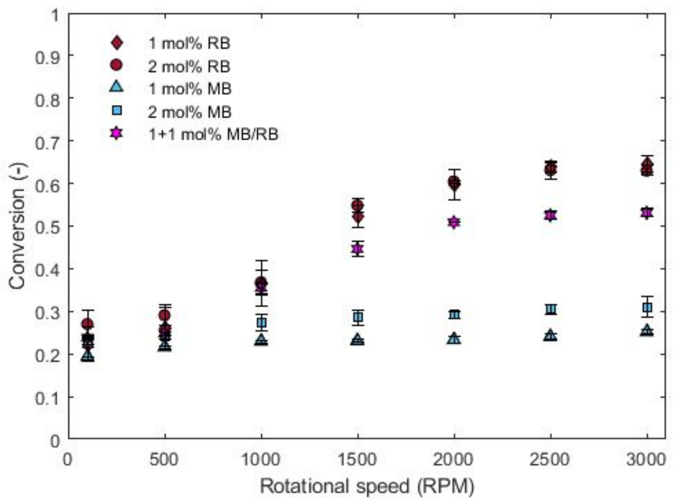

SI Figure 32: The effect of a mixed photocatalytic system on conversion tested in the pRS-SDR with an irradiance of 1.70 W cm^{-2}, a flowrate of 25 mL s^{-1}, an initial α-terpinene concentration of 0.1 M. The gas-liquid ratio was 3:1.

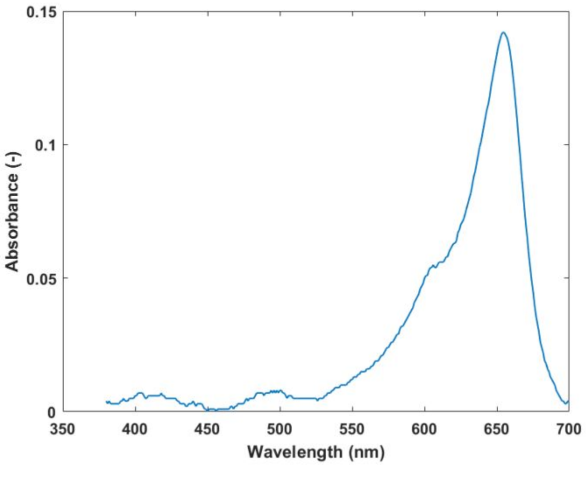

SI Figure 33: The measured absorption spectrum of methylene blue photocatalyst (C_{MB} = 7.84 x 10^{-4} M) which was measured using a offline UV-Vis.

7. Calculation Details

7.1 Relevant Optical Output

A threshold of 10% of the normalized (to peak) absorption of Rose Bengal is used as an arbitrary cutoff to distinguish the wavelength regions for absorption of light by the photocatalyst. Based on this analysis, the Radiant Flux curve (SI Figure 1) is integrated in the relevant wavelength range to determine the relevant optical output with regards to the light. The analysis is carried out using MATLAB.

7.2 Optical Flux

The optical flux (W cm^{-2}) is calculated by taking the relevant optical output at a particular dimming voltage (W) and dividing it by the area of the top stator surface (diameter=134 mm).

$$\text{Optical Flux} = \frac{\text{Relevant Optical Output (W)}}{\text{Area of top stator}(\text{cm}^2)}$$

(SI Eq.1)

7.3 Calculation of Conversion

The samples obtained at the exit of the reactor were analyzed with GC-FID and the relevant calibration curve was used to determine the concentration of starting material left. The conversion was obtained from the equation shown below, where the initial concentration of starting material was determined from the GC-FID analysis of the stock solution prepared for the particular experiment.

$$\text{Conversion (X)} = \frac{C_{\text{inital}} - C_{\text{final}}}{C_{\text{inital}}}$$

(SI Eq.2)

7.4 Calculation of Productivity

The calculation for the productivity (based on the conversion of starting material) is shown in the equation below. The factor 86.4 is for the conversion from g s^{-1} to kg day^{-1}.

$$\text{Productivity}\left(\text{kg day}^{-1}\right) = Q_L \times X \times C_{SM,0} \times MW_{SM} \times 86.4$$

Q_L= Volumetric Flow Rate (L s^{-1})

X= Conversion (-)

$C_{SM,0}$= Concentration of starting material inlet flow (mol L^{-1})

MW_{SM}= Molecular Weight of starting material (g mol^{-1})

(SI Eq.3)

7.5 Calculation of Energy Dissipation Rate

The energy dissipated by the light source is calculated based on the energy used at the highest irradiance value (1750 W) and multiplying it by the fraction of the voltage (max voltage of 8 V).

$$E_{\text{Light}}(\text{W}) = 1750 \times \frac{\text{Voltage}}{8\ \text{V}}$$

(SI Eq.4)

The energy dissipated by the pRS-SDR is based on the correlation described in our previous study[1].

$$E_{pRS-SDR}(W) = 5.73 \times 10^{-12}\ h^{-0.14}{Re_{\omega}}^{2.12}$$

Re_{ω}= Rotational Reynold Number (-)

h= Gap Ratio (-)

(SI Eq.5)

The rotational Reynolds number can be calculated through the following expression

$$Re_{\omega}(-) = \frac{\omega r^2}{\vartheta}$$

ω= Rotation Speed (rad/s)

r= Rotor Radius (m)

ν= Kinematic Viscosity (m^2s^{-1})

(SI Eq.6)

The gap ratio can be calculated as

$$h(-) = \frac{g}{r}$$

g= rotor stator gap (m)

r= Rotor Radius (m)

(SI Eq.7)

The total energy dissipated by the system was then just taken as a sum of energy expended by the light source and the energy expended by the pRS-SDR.

References

(1) Chaudhuri, A.; Kuijpers, K. P. L.; Hendrix, R. B. J.; Shivaprasad, P.; Hacking, J. A.; Emanuelsson, E. A. C.; Noël, T.; van der Schaaf, J. Process Intensification of a Photochemical Oxidation Reaction Using a Rotor-Stator Spinning Disk Reactor: A Strategy for Scale Up. *Chemical Engineering Journal* **2020**, *400* (June), 125875. https://doi.org/10.1016/j.cej.2020.125875.
